# Nuclear Mechanics and Nuclear Mechanotransduction in Cancer Cell Migration and Invasion

**DOI:** 10.3390/biom16030457

**Published:** 2026-03-18

**Authors:** Claudia Tanja Mierke

**Affiliations:** Faculty of Physics and Earth System Sciences, Peter Debye Institute of Soft Matter Physics, Biological Physics Division, Leipzig University, 04103 Leipzig, Germany; ctmierke@gmail.com

**Keywords:** softness/stiffness, nesprins, lamins, metastasis, mechanobiology, forces, viscoelasticity, mechanosensing, mechanotransdcution, nuclear pore complex

## Abstract

Nuclear mechanics and mechanotransduction are involved in the migration and invasion process, such as those in which the cells need to deform themselves to pass through constrictions. Specifically, properties like nuclear softness, viscoelasticity, plasticity (like nuclear pore complexes) and deformability are critical in cancer and its malignant progression. The nucleus represents a physical barrier for the migration and invasion in dense 3D extracellular matrix (ECM) scaffolds. Therefore, the deformability of the nucleus seems to determine the migration limit in circumstances where the enzymatic remodeling of the surroundings is impaired. There are still significant knowledge gaps regarding effects of nuclear deformation during cancer dissemination. It seems that nuclear deformation can alter gene transcription, induce alternative splicing processes, impact nuclear envelope rupture, nuclear pore complex dilatation, damage the DNA, and increase the genomic instability. These mechanically induced alterations can in turn impact the migratory behavior of the cancer cells. The stiffness of the nucleus relies on the condensation of chromatin, and the nuclear lamina, which consists of a network of intermediate filaments underneath the nuclear envelope. All of this is discussed in the review and it is argued that nuclear deformability is universally found in various cancer types. Another focus is placed on the nuclear envelope proteins like emerin, and the SUN-KASH complex and how they contribute to the Linker of Nucleoskeleton and Cytoskeleton (LINC) complex, which consequently couples the nucleus and the cytoskeleton. It is argued that this connection is crucial for force transmission, which governs nuclear stiffness dynamically, depending on the force applied. In this review, recent findings are described that couple ECM-induced nuclear mechanosensing and mechanotransduction with the migration and invasion of cancer cells. Moreover, it is suspected that changes in the mechanosensory characteristics of the cell nucleus could play a pivotal part in the malignancy of cancer cells and the heterogeneity of tumors. Finally, it is discussed what impact the individual elements of the nucleus offer to mechanically alter cellular migration and invasion in cancer and its malignant progression.

## 1. Introduction to Mechanosensation, Mechanotransduction and Nuclear Mechanics

**Figure 1:**
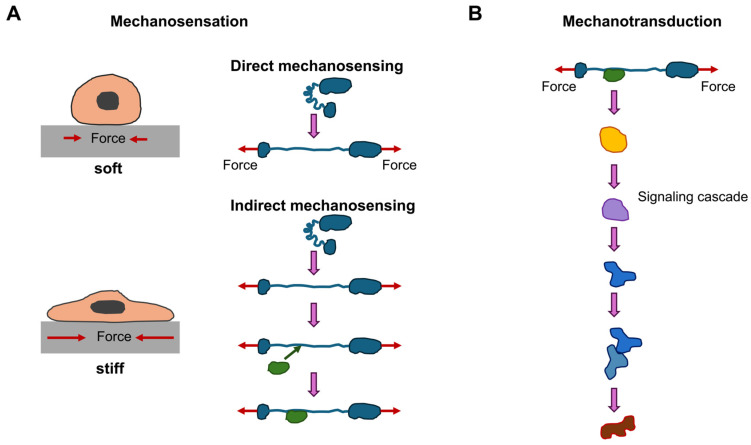
The schematic illustration depicts the definitions of mechanosensory and mechanotransduction. Despite these commonly used terms, which pertain to mechanical interplay within cells, being used synonymously, they possess separate meanings. (**A**, top) “Mechanosensitive” covers the broadest range of meanings and is a general term for all direct and indirect mechanosensing and mechanotransduction events. Cell spreading, which relies on the stiffness of the substrate, is an illustration of mechanosensitive conduct, encompassing both mechanosensory perception and subsequent mechanotransduction. (**A**, bottom) “Mechanosensing” specifically refers to the reaction to an exerted force, which occurs either directly (e.g., a protein that undergoes a conformational shift in reaction to an exerted force; blue) or indirectly (e.g., a protein that detects a binding site on another protein that has been exposed by the exerted force; green). (**B**) “Mechanotransduction” refers to the conversion of a mechanical input like stretch, shear stress or pressure into a biochemical signal that initiates internal downstream processes.

Mechanobiological aspects are playing an increasingly important role in many physiological and pathological processes such as cancer, and their significance has become particularly evident in the migration and invasion of cancer cells. Many new terms are used in the field of mechanobiology and related areas, whose meaning is not so familiar to scientists from classical biological, physical and medical disciplines. Therefore, the terms “mechanosensing” and “mechanotransduction” must first be clearly defined, as they are often used synonymously, even though they are quite distinct from one another. This can lead to confusion and misunderstanding. By precisely defining the terms, they can be used appropriately in each case, so that the differences become apparent. Both terms, mechanosensing and mechanosensitivity, generally refer to a process that is directly or indirectly altered by forces acting on a living cell (Figure 1).

In contrast, mechanosensory perception is defined as the direct effect of a force that leads to a change in behavior (i.e., a reaction). Indirect mechanosensory perception (i.e., mechanoreactivity) is defined as the reaction to a change in force exerted on another structure. Mechanosensory perception is therefore the first step in mechanotransduction. Using this concept, a process identified as mechanosensitive could actually be a consequence of the application of force and not directly participate in mechanosensory perception [1]. There are two simple examples. First, take a look at the cell spreading process, which intuitively and easily illustrates how mechanical alterations in the surrounding environment during normal development or under disease conditions can profoundly influence cell functions [2,3]. When otherwise identical conditions apply, cells that are seeded onto soft substrates have a tendency to be spherical and spread out substantially less compared to cells on stiff substrates, where the cells tend to adopt a flattened shape and generally become polarized [4]. According to the definition, the process of cell spreading is mechanosensitive because it relies on the mechanical characteristics of the underlying support.

The actual mechanosensory activity that takes place in the propagation process is accomplished by a set of integrins, which comprise a varied family of heterodimers that traverse the plasma membrane and link the internal cytoskeleton with the extracellular matrix (ECM), and a variety of proteins within focal adhesions (FAs) [5,6,7]. Both processes are triggered by forces produced in the actomyosin cytoskeleton. Although the functional process of spreading is unambiguously mechanosensitive, it is a consequence of these mechanosensitive events. Beyond cell spreading based on substrate or matrix stiffness, there is another example of mechanosensing, such as cells display durotaxis behavior as they crawl from soft to stiff substrates. The process has been shown to rely on the polarization of non-muscle myosin-II, the B isoform (MIIB) [8,9]. Nevertheless, non-muscle myosin II, the A isoform (MIIA), plays an crucial upstream function: in cells on a soft matrix, MIIA showed up as diffuse and moving, while on a stiff matrix it stuck together tightly in aligned stress fibers, which were subsequently polarized through MIIB [8]. MIIB seems to be crucial for the persistent movement of cells on durotactic matrix gradients, and the degree of MIIA phosphorylation can influence durotaxis as well as the polarization of the cytoskeleton [10]. In the opposite case, there can also be reverse durotaxis [10].

Lastly, mechanotransduction is the conversion of mechanical stimuli into biochemical stimuli and, per definition, is a process that follows a mechanosensory event. Certain proteins, like the focal adhesion proteins talin1 (hereafter denoted as talin) and vinculin, which couple the actin cytoskeleton to the ECM via integrins, perform multiple functions and can be involved both directly in mechanosensory perception and indirectly in subsequent mechanotransduction events. Mechanotransduction alone can encompass a number of proteins and signaling processes, or it can be started by a sole protein. For example, the protein zyxin from the LIM domain family utilizes its C-terminal LIM domains to detect stretched actin filaments within stress fiber fractures and begins their repairing process through its N-terminal vasodilator-stimulated phosphoprotein (VASP) and α-actinin binding domains [11,12]. Consequently, the sustenance of stress fibers is turned into a sharply localized mechanotransduction event. In sharp opposition, the stretching of talin that accompanies cell adhesion comprises several signaling steps, including alterations in the interactions between talin, vinculin, the Rap1 GTP-interacting adaptor molecule (RIAM, alternatively referred to as Amyloid beta precursor protein binding family B member interacting protein (APBB1IP)), and their linked downstream associates, like talin or vinculin [13,14]. In any case, however, there is a certain critical point that triggers the transformation of force into a modification of the biochemical cues.

Direct mechanosensory perception was presented above, which raises the question of what is meant by indirect mechanosensory perception. The direct mechanosensing processes presented refer to a force that acts directly on the cell’s proteins, thereby altering their conformation and biochemical activity. A different group of mechanosensitive proteins is affected in its activity by the force exerted on its binding proteins. They therefore do not correspond to the definition of mechanosensory systems outlined above, as they are merely indirectly influenced by variations in force. Even though these mechanoresponsive phenomena may technically be termed mechanotransduction, it can be argued that these indirect mechanosensing processes deserve a separate topic of discussion.

Strain sensing is most commonly linked to members of the LIM domain protein family, which comprises a range of mechanosensors [15]. LIM domains consist of two zinc finger motifs that enable a variety of protein–protein interactions, and a lot of members of this protein family feature several LIM domains [16]. The protein zyxin from the LIM domain family was initially recognized as a focal adhesion protein that shifts from the adhesions to the actin stress fibers in reaction to cyclic stretching [17]. Follow-up studies have demonstrated that Zyxin also temporarily migrates to spontaneous tears in stress fibers to facilitate their repair [12]. Notably, the three LIM domains in the C-terminal half of zyxin sense elongated (i.e., stretched) actin filaments in the stress fiber, and reparation is achieved through the enlistment of alpha-actinin and the actin-regulating proteins Mena (also called enabled homolog (ENAH)) and vasodilator-stimulated phosphoprotein (VASP), both of which attach to the N-terminal portion of zyxin. Later research has shown that other LIM domain proteins act in a similar way, like Hydrogen Peroxide–Inducible Clone 5 (Hic-5, synonymously referred to as transforming growth factor beta-1 induced transcript 1 (TGFB1I1)) and Cysteine and Glycine-Rich Protein 2 (CRP2, synonymously referred to as CSRP2) [18], paxillin [19], four-and-a-half LIM domains protein 2 (FHL2) [20], engima (synonymously referred to as PDZ and LIM domain 7 (PDLIM7)) [21], and testin [22].

Indirect mechanosensory detection comprises the perception of mechanical signals by secondary cells or structures, whereby cells utilize ion channels (such as Piezo1) that undergo physical stretching or deflection due to applied forces and convert physical stresses into electrical impulses [23,24]. These channels serve as direct transducers that convert mechanical stress into ion flow (such as Ca^2+^ influx) and thereby into electrical signals that are vital for various physiological functions, such as control of blood pressure and maintenance of cell volume. Some cells utilize direct channels (such as Piezo) whereas others employ secondary structures or linkages (connections to the ECM/cytoskeleton) to convey force, although the result is analogous: the conversion of physical stimuli into cellular reactions. What is the network of mechanosensing cellular elements? It consists of cell surface receptors, focal adhesion complexes, cytoskeletal elements and complexes, organelles such as mitochondria, and cell nuclei.

Why is the cell nucleus so important? While mechanosensory and mechanotransduction processes are well documented at the cellular level, these mechanisms are not yet as thoroughly characterized and discussed at the level of cell organelles such as the cell nucleus. Besides its canonical features in genetics and molecular biology, the cell nucleus performs an extremely significant part in the perception and reaction to mechanical stimuli during a process referred to as nuclear mechanotransduction. As the largest organelle in the cell, the cell nucleus plays a particularly important role in the migration and invasion of cancer cells through the dense extracellular networks of their three-dimensional environment, where the constrictions are smaller than the cell diameter, whereby the mechanical properties of the cell nucleus and its mechanical adaptability are especially effective. This becomes particularly noticeable when ECM-degrading enzymes play a minor part and are either absent or present only in small quantities. Therefore, the importance of the cell nucleus in mechanotransduction and the mechanical characteristics of the cell nucleus are presented and discussed in terms of their significance in the migration and invasion of cancer cells. Special focus is placed on the viscoelasticity of the cell nucleus, although most studies analyze and discuss the stiffness or softness of the cell nucleus that controls the migration and invasion of cancer cells during cancer metastasis. Beyond the elements or structures of the cell nucleus that contribute to its mechanical properties, the effect of mechanotransduction in relation to the induction of alternative splicing and the regulation of gene expression is discussed.

## 2. The Mechanosensory and Mechanotransduction System

Living cells and cell nuclei operate in such a tightly integrated manner that mechanical traction on the receptors on the cell surface can instantly alter the organization of molecular structures within the cytoplasm and cell nucleus. This means that both whole living cells and cell nuclei are hard-wired [25]. The term hard-wired also applies to cytoskeletal elements comprising actin filaments, intermediate filaments, and microtubules. Each of these plays an interconnected role in transferring mechanical stress and triggers the dynamic remodeling at the molecular level, which is referred to as tensegrity model.

### 2.1. The Tensegrity Model Proposes a Coupling Between Environment, Cytoskeleton and Nucleus

As the integrins were pulled through micromanipulation of tethered microspheres using magnetic tweezers or micropipette aspiration techniques, the cytoskeletal filaments realigned, the cell nuclei deformed, and the nucleoli repositioned themselves parallel to the axis of the exerted stress field [26]. These responses were unique to integrins, not affected by changes in the cortical membrane, and caused by direct connections between the cytoskeleton and the cell nucleus. The diverse cytoskeletal filaments, like actin filaments, intermediate filaments, and microtubules, fulfill distinct functions. Actin filaments facilitate the propagation of force in the cell nucleus under low strain; when subjected to more severe deformation, nevertheless, the actin network ruptured. In the opposite scenario, intermediate filaments successfully transmitted force to the nucleus in both situations. The actin and intermediate filament networks also served as molecular restraints to mechanically stiffen and tether the cell nucleus in its location, while microtubules served to keep the intermediate filament lattice open and secure the cell nucleus resistant to sideways compression. Molecular bridges between integrins, cytoskeletal filaments, and nuclear skeletons represent a specific pathway for mechanical signal transduction within cells and a mechanism for generating integrated alterations in cell and nuclear architecture in reaction to alterations in the adhesiveness or mechanics of the ECM scaffold [25]. It is crucial to examine how mechanical stresses acting on the plasma membrane can drive coordinated alterations in cell, cytoskeleton, and nuclear shape, since this could provide valuable insights into mechanotransduction. The integrated regulation of cell shape appears to be achieved through the “hard-wiring” of transmembrane ECM receptors, cytoskeletal filaments, and nuclear scaffolding, such that mechanical tension on the plasma membrane leads to a concerted reorientation of structural constituents within this interconnected molecular architecture [26,27]. This model strongly contrasts with many existing models of basic cell mechanics, which consider the viscous, fluid-like cytoplasm and the enclosing elastic membrane to be the most important supporting structures inside living cells [28,29,30]. Among these models are the best-known continuum models or viscoelastic models, which frequently incorporate features such as series-connected spring-damper elements within Maxwell models or parallel-connected spring-damper elements in Voigt models to account for the mixed liquid-like flow and solid-like elastic behavior of the cell. The concepts of the fluid-elastic models are as follows. First, the viscoelastic model: Living cells are neither purely elastic (solid) nor purely viscous (liquid), but possess both characteristics, which means they absorb and release energy and undergo deformation over time when subjected to stress. Second, in the Maxwell model, the cells are depicted as a spring (elasticity) and a damper (viscosity) in serial arrangement, which illustrates an immediate elastic response accompanied by a viscous flow. Third, in the Voigt model, a spring and a damper are coupled in the parallel configuration, which results in solid-like characteristics over longer periods of time in which the displacement is directly commensurate with the applied force. Fourth, in a continuum mechanics model, the cell is considered as a continuous substance, with an emphasis on volume characteristics such as stiffness and viscosity, which are appropriate for deformations on a larger size scale. In addition to physical considerations, microscopic examinations reveal structural connectivity between ECM molecules, transmembrane proteins, cytoskeletal filaments, and nuclear scaffolds present in cells that have been extracted with the use of a detergent [31,32,33]. All of which supports the hard-wired model that seems to be based on genetically set systems, but there seems to be also a certain degree of fine-tuning based on experience, which is referred to as cellular or nuclear plasticity.

Quantitative analysis of the mechanical characteristics of the cytoplasm and nucleus revealed that their structural interaction within the cytoskeleton is complicated and follows nonlinear mechanics, meaning that their reaction to force is disproportionate and acts more like active gels instead of simple elastic solids, which is crucial for comprehending cell mechanics. The performance of these various filament systems is not straightforwardly cumulative or superimposable. Overall, the cytoskeletal filament system operates as a highly integrated, synergistic framework rather than a collection of independent elements. Actin filaments create a gel that fills space and handles pressure effectively, but it lacks the stiffness to handle outside tension and breaks under heavy loads. The intermediate filament matrix itself has low resistance to lateral compressive stress, but it effectively withstands tension and stiffens under heavy strains. Similar results were achieved by examining purified filament networks in vitro [34]. Nevertheless, when these two filament networks are incorporated in living cells, a higher-order composite structure emerges that fulfills lead-bearing functions in a more efficient manner. Complete mechanical reactiveness and structural robustness, nonetheless, additionally necessitate the participation of microtubules to locally oppose the inward contraction of the encircling tensile cytoskeleton, thus exerting an internal tension or “prestress” within this interlinked molecular framework. Moreover, vimentin augments the amount of stable acetylated microtubules [35]. Cytoplasmic actin filaments and intermediate filaments also seem to act as tension anchors, holding the cell nucleus in position, coordinating alterations in cell and nucleus shape, and imparting mechanical stiffness to the cell nucleus.

Dependence on distinct load-bearing components, tensile continuity, and prestressing for form stability aligns with a model of cell and tissue architecture founded on tensegrity architecture [26]. What does the concept of tensegrity mean? Tensegrity is a term used to describe network configurations that are mechanically stabilized due to the presence of tension. Tensegrity is a term used to describe network configurations that are mechanically stabilized due to the presence of tension. They consist of tensioned components that draw towards the midpoint and are counterbalanced by other components that oppose compression. The outcome is a robust and resistant system that reacts to environmental mechanical forces. Tensegrity explains how local stresses can cause coordinated alterations in the cellular, cytoskeletal, and nuclear architecture without involving protein polymerization or diffusion-based signal transduction [27], and how various types of cytoskeletal filaments can uniquely influence the overall mechanical response of the cell. It also offers a mathematical foundation for forecasting the material characteristics and architectural traits of living cells, regardless of alterations in cytoskeletal linkages [26,36]. This contrasts with percolation theory, which is a mathematical approach to analyzing the significance of phase transitions and network interconnectivity [37]. Tensegrity offers a mathematical foundation for form stability [36], whereas percolation delivers a complementary method for characterizing how the mechanical properties of tensegrity-based frameworks may vary in reaction to modifications in polymerization or cross-linking of the cytoskeleton. The “hard-wired” aspect ultimately pertains to how these essential components are genetically and physically interconnected to preserve cellular integrity and react to mechanical stimuli, thereby transforming the cell into a self-stabilizing, force-balancing structure. While the tensegrity model highlights the cell’s intrinsic structural framework, fluid-elastic models primarily consider the cytoplasm and membrane as key constituents, reflecting the dynamic, time-dependent mechanical characteristics of the cell.

### 2.2. What Causes the Collective Nonlinear Behavior of Cytoskeletal Networks?

There are four major factors that contribute to their nonlinear characteristics. First, there exists mechanical synergy and a stiffening behavior. For instance, introducing a small quantity of microtubules into an actin meshwork results in substantial, nonlinear elastic stiffening, as the stiff microtubules counteract uneven stretching in the more flexible (softer) actin filaments. As a result, the mechanical characteristics of a mixed filament system frequently surpass the summation of its individual building blocks. Second, there is a physical interrelationship and interaction between the three filament systems. The filament systems actively control each other using direct molecular connections like plectin or adenomatous polyposis coli (APC), and motor proteins, such as kinesin, dynein, and myosin. As a result, the cytoskeleton is stabilized through intermediate filaments such as vimentin, that stabilize microtubules from depolymerization and can assist in their “rescue.” Filament systems can serve as physical barriers, as dense actin scaffolds restrict the growth of microtubules and promote their turnover. Thirdly, there exists regulatory feedback circuits. The systems communicate through signal transduction cascades, where the dynamic behavior of one filament type causes biochemical modifications within other filament types. For example, the lengthening and shortening of microtubules stimulates Rac1 and RhoA signal transduction, thereby driving the polarization and formation of the actin cytoskeleton necessary for cell motility. Fourth, there exist competitive dynamics. These filaments of the cytoskeleton commonly fight over shared sources like monomers or accessory proteins [38]. These “competing interactions” determine the overall structural organization and function of the cell, implying that the ultimate state of the cell cannot be accurately anticipated by merely summing the individual behaviors of each filament network in its isolated state [38]. Intermediate filaments like vimentin build viscoelastic web-like networks [34] which are assumed to limit the dynamics of the cytoplasm [39] and the bending modes of microtubules [40]. Nevertheless, the mechanism of this repression and its effects on the cell-wide microtubule meshwork are scarcely comprehended and have major consequences for polarization, motility, and the traction forces generated by the cells [35,41]. Particular attention has been paid to the effects of depletion of vimentin intermediate filaments (vim^−/−^) and blocking actomyosin activity or triggering depolymerization of actinin. Through spatial image autocorrelation analysis, it has been observed that depletion of vimentin reduces the effective mesh size of the microtubule framework, independent of the state of actin, whereas blocking myosin activity or causing actin depolymerization enhances the mesh size of microtubule framework in cells whether or not vimentin is present. In addition, differential dynamic microscopy has revealed that microtubules display superdiffusive motility over a broad spectrum of length and time scales, with increased velocity in all cells depleted of vimentin and impaired velocity under conditions of aberrant actin organization. These findings point to opposing and apparently independent functions that vimentin and actin perform in shaping the dynamism and architecture of the microtubule system in cells over several decades of time and space. These effects could be due to the framework-forming capacity of vimentin and the dynamics of active actin, which separately control the dynamics and organization of the microtubule framework in cells [38].

### 2.3. Focal Adhesion Complexes at the Cell Membrane Serve as Hubs for Mechanosensing

**Figure 2:**
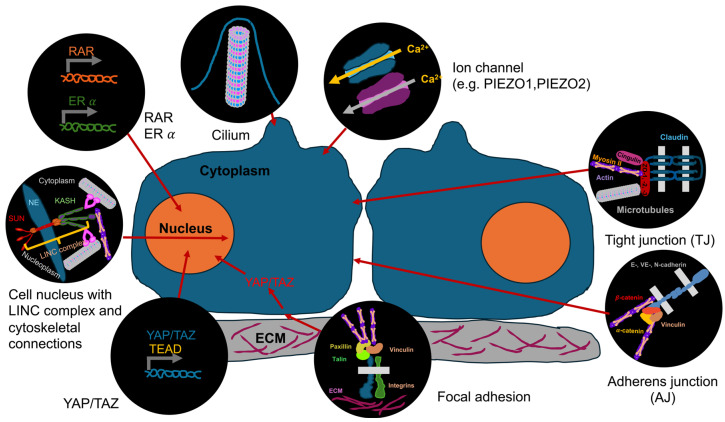
External environment, cytoskeleton and cell nucleus are coupled via a physical connection. Environmental mechanical cues like forces can trigger the activation of ion channels, such as PIEZO1 and PIEZO2, which causes an influx of Ca^2+^ ions into the cytoplasm. ECM scaffold binding adhesion receptors like integrins are subject to external forces that are transmitted via FAs to the actomyosin cytoskeleton into the cell nucleus. This causes YAP/TAZ to move into the cell nucleus, where it interfaces with the transcription factor TEAD and subsequently activates gene expression. Similarly, RAR and ER α are able to translocate into the cell nucleus, where they induce gene transcription. Neighboring cells interact through TJs and AJs that are coupled to the cell’s cytoskeleton that is composed of actin-filament lined to myosin filaments, microtubules and intermediate filaments. These cytoskeletal structures are connected to the nuclear elements via the LINC complex. Certain cell types compromise cilia that can be mechanically perturbed and thereby induce a mechanosensing and mechanotransduction process via cytoskeletal filaments like microtubules.

In adherent cells, integrins attach the cell to the ECM via FAs (Figure 2). Under the influence of forces, integrins experience conformational alterations that lead to more robust catch bonds to the ECM [42], resulting in the development and maturation of FA, which consist of adapter proteins, kinases, and various other signaling modules (Figure 2). Via adapter proteins like α-actin, talin, and vinculin, FA attach to the actin cytoskeleton, which is the main load-bearing part of the cell. Cell–cell junction complexes like adherens junctions (AJs) and tight junctions (TJs) work as mechanosensors in the same way (Figure 2).

AJs are composed of transmembrane proteins from the cadherin family that interface with actin through catenins. When subjected to tension, α-catenin undergoes conformational rearrangement, exposing a cryptic vinculin-binding domain that promotes vinculin recruitment to the AJ and promotes the binding of actin [43]. TJs are composed of transmembrane proteins from the claudin and occludin families, which are connected to actin and microtubules using adapter proteins (Figure 2). The actin cytoskeleton has a high dynamic range and is a polymorphic network that quickly adapts to biomechanical challenges [44].

Mechanical signals can be perceived by cells via a wide range of membrane-anchored receptors, among them are direct sensors like strain-activated ion channels, for instance Piezo1/2, and transient receptor potential (TRP) channels like transient receptor potential vanilloid 4 (TRPV4), Histamine H1 receptor (H1R) [23,45], and indirect ones like, integrins, and cadherins as well as combined direct and indirect sensors like G protein-coupled receptors (GPCRs) that span the plasma membrane [46,47,48]. GPCRs can be activated directly through force, as in the case of GPR68, or they can be coupled to components of the cytoskeleton, thereby associating mechanical influences with G protein signal transduction. Another example of directly mechanically activated GPCRs is the angiotensin II type 1 receptor (AT1R), which perceives mechanical stress and participates in the control of blood pressure. These receptors become ligand independently activated by mechanical cues. Distinct to ligand-independent regulation of GPCRs is the ligand-dependent indirect activation via mechanical signals. Specifically, paracrine signaling can occur because mechanical stimulation triggers cells to secrete chemical messengers (ligands) or lipids like prostaglandins, which subsequently activate nearby GPCRs such as GPR120 and GPR109a, thereby coupling physical forces with chemical signaling processes. Another mechanism involves the integration of both mechanical and chemical inputs into GPCRs, which occasionally results in distorted signal transduction, such as discrepancies in responses to the activation of the identical receptor.

The clearly indirect mechanical activation proceeds in integrin- and cadherin-based adhesion complexes, which form at the contact sites between the cell and the ECM or between cells. Both are composed of proteins that are sensitively responsive to alterations in tensile forces and adjust their arrangement and dynamics as a result, leading to biochemical responses that propagate mechanical impulses [49,50]. Forces between the external milieu and the intracellular actin cytoskeleton are predominantly conveyed across integrin-containing focal adhesions and cadherin-containing AJs. The interaction between these complexes is widely acknowledged and influences the mechanical features of the cell. Nevertheless, integrins and cadherins form large families of adhesion receptors and interact with a variety of ligands, adapter proteins, and cytoskeletal filaments to generate multiple assemblies. Recent findings suggest that integrin-containing hemidesmosomes counteract force transmission and the generation of traction forces through focal adhesions to preserve cellular stability [51]. The cytolinker protein plectin facilitates this interplay by connecting intermediate filaments to the actin cytoskeleton [52,53]. Likewise, cadherins in desmosomes could regulate the force generated through adhesive connections. In addition, mechanotransduction can be affected through mechanosensitive structures, such as podosomes, clathrin lattices, and tetraspanin-enriched microdomains. Podosomes are an actin-rich structural element that function as active mechanotransducers responsible for assessing substrate stiffness [54]. Podosomes are known to exploit actin polymerization and myosin II-facilitated contractility to perceive and react to the elasticity of the matrix and geometric signals [55]. Clathrin lattices are frequently termed “reticular adhesions” or “plaques.” These flat clathrin lattices serve as contractility-independent mechanosensors. They form in reaction to a high substrate stiffness due to “frustrated endocytosis,” in which like the αvβ5 integrin attach to the surface scaffold, thereby blocking the formation of vesicles and, instead, encouraging intracellular signal transduction, such as the activation of Erk [56]. Tetraspanin-enriched microdomains (TEMs) refer to domains that serve as “building blocks” for intricate membrane architectures including migrasomes and digitation junctions (DJs), which are highly specialized cellular architectures that facilitate cell–cell or cell–substrate communication [57]. DJs are rich in tetraspanins such as CD9, CD81, and CD82, along with associated molecules like integrin α3β1, CD44, and EWI-2/prostaglandin receptor-like (PGRL). Migrasomes are newly identified organelles generated from migrating cells that are critical for cell-to-cell communication. They originate at the ends or branches of retraction fibers and transfer cellular signals and material to neighboring cells [58]. TEMs segregate signaling proteins and adhesion receptors, control their affinity, and promote signal transduction following cell-to-cell and cell-to-matrix exchanges.

### 2.4. Cytoskeletal Elements Process Mechanosensory Cues in the Cytoskeleton

The concept of cellular mechanosensors has been used to describe a set of molecules, primarily proteins, that undergo a state alteration in reaction to mechanical input. The type and extent of alterations induced by mechanical signals can differ substantially. Among the mechanical alterations are post-translational modifications, such as p130Cas [59], paxillin [60,61,62], cofilin [63], focal adhesion kinase (FAK)-controlled yes-associated protein (YAP) activation [64,65], and nuclear lamin A/C [66], intracellular shuttling, such as β-catenin [67,68], zonula occludens-1 [69], c-Abl [70], zyxin [71], and armadillo [72], protein unfolding, like stretching of talin [13,65], and the emergence of novel interactions, such as the binding of vinculin to talin and actin filaments [73] that are regarded as positive signs of mechanical responsive ability.

Specifically, cytoskeletal elements, such as stress fibers can be affected by mechanical cues. For instance, stress fiber structures rapidly change in reaction to alterations in the biophysical characteristics of the cellular microenvironment. The maturation of stress fibers relies on the mechanosensitive stimulation of 5′-AMP-activated protein kinase (AMPK), which then phosphorylates VASP to block the polymerization of actin at focal adhesions. Ca^2+^-calmodulin-dependent kinase kinase 2 (CaMKK2) has been found to be a key upstream regulator of mechanosensitive activation of AMPK [74]. CaMKK2 and Ca^2+^ inflows were concentrated within focal adhesions near the tips of contractile stress fibers. Blocking CaMKK2 or mechanosensitive Ca^2+^ channels caused abnormalities in the phosphorylation of AMPK and VASP, leading to a breakdown of contractile bundles and a reduction in the forces exerted by the cells [74]. These observations demonstrate that Ca^2+^, CaMKK2, AMPK, and VASP constitute a mechanosensitive cascading pathway in focal adhesions that is key to the development of stress fibers.

According to their protein constitution and their attachment to focal adhesions, stress fibers can be classified into three subcategories [75,76]. These comprise non-contractile “dorsal stress fibers,” slender “transverse arches” coursing retrograde to the cell midpoint, and “ventral stress fibers,” which consist of thick actin-myosin bundles formed by the union of several slender transverse arches as they move in a centripetal direction. In addition, the ventral stress fibers constitute the key force-sensitive and force-generating actomyosin bundles present in crawling and invading cells [77,78,79,80]. The merging of the transverse arcs and the accompanying generation of ventral stress fibers are linked to an elevation in contractile force [80,81]. It has been hypothesized that there are far-reaching attractive forces operating between separate myosin II filaments while they are assembling into stacks. Therefore, the merging of transverse arcs and the assembly of myosin II stacks occur simultaneously when stress fibers are created [82,83,84,85]. Throughout centripetal flow, transverse actin arcs join together, along with the building of myosin II assemblies, to develop mechanically sensitive actomyosin bundles [86]. Consequently, the arrangement and directional organization of actomyosin bundles are subject to accurate mechanosensitive guidance. Their maturation process is dependent on a mechanosensitive inflow of Ca^2+^ and the subsequent activation of the Ca^2+^/calmodulin-dependent kinase kinase-2 (CaMKK2)-AMP-activated protein kinase (AMPK)-VASP signaling network, as is the case in human osteosarcoma cells [74,80]. Nevertheless, the complete role of myosin II stacking in cancer progression remains unclear, as there are no specific techniques to specifically interfere with myosin II stacking. For instance, it is unknown whether integrated myosin II stacking is necessary for the control of the mechanical-sensitive arrangement of contractile stress fibers. Expression levels are implicated in the advancement of several types of cancer, among them colorectal cancer [87], liver cancer [88], lung cancer [89] and ovarian cancer [90]. Various stress fiber-crosslinking proteins, such as α-actinin, filamin, and non-muscle myosin IIB (NMMIIB), are involved in stress fiber elasticity in living melanoma, myoblast and osteosarcoma cells [91,92,93]. In addition, it is established that in human migratory osteosarcoma cells, deprivation of myosin-18B selectively suppresses the assembly of myosin-II stacks and the associated subsequent maturation of contractile actin stress fibers [94]. Consequently, myosin-18B offers an efficacious approach to investigate the physiological relevance of myosin II stacking [86]. Myosin-18B has been found to be key to the mechanosensitive CaMKK2-AMPK-VASP signal transduction chain involving contractile actin stress fibers [86]. Myosin-18B is an unconventional myosin of class XVIII, and was initially found to be a tumor suppressor [89]. The myosin-18 family distinguishes itself from traditional myosins like myosin II in that it often lacks motor ATPase activity [95]. As a result, there is growing evidence indicating mutation in the myosin-18B gene and its altered myosin-18D promote the mechanosensitive CaMKK2-AMPK-VASP control of contractile actin stress fibers. Ultimately, contractile stress fibers, at least in osteosarcoma cells, are incapable of sustaining tension or thickening when myosin-18B is not expressed [86]. Overall, these results deliver crucial new knowledge about the physiological relevance of higher-order myosin II arrangements in cells and reveal their connection to the control of cell mechanics [86]. Myosin-18B has a key function in mechanotransduction and operates as a structural gluing agent that aids in the building and strengthening of myosin-II stacks within contractile actin stress fibers, which serve as critical components for perceiving and reacting to mechanical forces. Its deficiency perturbs these stress fibers, causing impaired cell motility, faulty mechanical force production, and attenuation of signaling cascades, such as the CaMKK2-AMPK-VASP pathway, demonstrating its function in the conversion of mechanical signals into cellular events including proliferation and migration. Myosin-18B acts as a building block that permits the actin cytoskeleton to operate in a force-sensing capacity [96].

### 2.5. Mechanical Coupling of Focal Adhesions, the Cytoskeleton and the Nucleus

Physical coupling from the cell-adhesion molecular complexes to the nucleus via the cytoskeleton has been identified. First of all, there is mechanotransduction at the plasma membrane [97]. Mechanical stimuli encompass shear stress, pressure, stiffness/compliance, or stretch. Similarly to how the positioning, amount, and timing of biochemical cues control their impacts, the strength, orientation, and spatial and temporal features of mechanical cues also influence their impacts [98]. Mechanical signals arising from the ECM scaffold, plasma membrane, and cytoskeleton act together in a bidirectional interaction with biochemical signaling pathways inside the cell [99,100,101]. One aspect is that mechanical stimulation leads to intracellular biochemical reactions that modify signaling cascades, which may even propagate all the way to the cell nucleus, in which mechanical cues can trigger alterations in gene expression [102]. Another aspect is that intracellular biochemical cues control mechanical responses through changes in the ECM scaffold, cytoskeletal proteins, and proteins exposed to the plasma membrane surface. Beyond that, cells are able to produce force and thereby regulate the mechanical signal transduction of neighboring cells and the nearby ECM scaffold. As a result, physical forces in the cell cortex and plasma membrane, as well as intracellular biochemical cues, work together in an intricate interplay to perceive and adjust to mechanical cues [103,104].

The nucleus measures and reacts to the external constraints. Nuclear mechanotransduction involves specific processes initiated by structures connected to or contained within the cell nucleus that interact particularly with nuclear elements, notably chromatin directly. The NE isolates genomic DNA within the cytoplasm and controls the transportation of proteins in and out of the nucleus. Preserving the intactness of the NE throughout interphase is deemed pivotal. In cases of especially wide-ranging deformations, for example, the penetration of immune cells and the spread of cancer cells through repeated and momentary fractures of the NE as they pass through the confined area of the interstitium, mechanical stresses can physically harm the cell nucleus and the underlying genetic material [105,106,107]. The NE closes quickly throughout interphase, which is supported by elements of the endosomal sorting complexes required for transport (ESCRT) III membrane remodeling apparatus. In addition, mechanisms have been uncovered that enable cells to combat mechanical stress to avoid harm by adjusting the mechanical characteristics of the cell nucleus or its elements [108,109]. This overview deals with both facets of nuclear mechanotransduction whereby the impact of nuclear damage toward the genetic material induced via mechanotransduction and the impairment of damage through changes in mechanical features of the nucleus are discussed.

## 3. Traditional Characteristics of the Nucleus Are Used as Biomarkers for Cancer

There are classic characteristics of the cell nucleus, such as cell size, cell volume, cell shape, and the positioning of the nucleus, which have been used to assess the extent to which cancer cells are pathologically altered in the case of cancer. Aberrant size/volume and shape are associated with diseases, notably cancer, where these features are considered in diagnostic and staging procedures. Specifically, the position of the cell nucleus plays a crucial role in the migration of cancer cells through narrow tissue confinements. Thus, an aberrant positioning of the nucleus seems to be related to increased motility and malignant progression of cancer. Ultimately, the position of the cell nucleus and its dynamically repositing correlates with the capacity of cells to metastasize.

### 3.1. Nuclear Size

The size of the cell nucleus depends on the type of cell and type of organism. In most animal cells, the diameter of the cell nucleus is usually between 5 and 15 μm [110]. Cell nuclear size is governed by a number of different factors. In addition, the cell nucleus can serve as an intracellular measuring tape to gauge alterations in cell shape [111,112]. How individual cells decipher information about their geometric shape under mechanical stress and physical space limitations in their local environment has been widely unexplored. It has been demonstrated that the cell nucleus acts as a non-dissipative cell shape deformation measuring device, permitting cells to track changes in shape continuously over a period of seconds. The unfolding of the inner nuclear membrane (INM), along with the relative spatial orientation of the cell nucleus within the cell, delivers physical insights into the amplitude and nature of cell shape variations. This adaptively triggers a calcium-dependent mechanotransduction cascade that governs the degree of actomyosin contractility and plasticity of migration. These data provide evidence for the hypothesis that the cell nucleus constitutes a functional module for cellular own-body perception, permitting cells to detect alterations in shape so that they can adapt their behavior to match their microenvironment.

Evidence suggests that in individual cells, the size of the cell nucleus is not influenced by the amount of DNA [113,114,115], but is closely related to the volumetric capacity of the cytoplasm and the dynamic nature of nucleocytoplasmic trafficking [113,116]. The cell and nucleus size differs depending on the cell type within the identical species, and the DNA content of the nucleus also corresponds to the size of the cell [117]. In addition, the size of the cell nucleus is actively regulated by certain molecular constituents. During Drosophila’s early stages of embryonic development, for instance, the cell nucleus experiences microtubule-dependent alterations in shape, transitioning from spherical to ellipsoidal, which is coupled with a considerable rise in the length of the nucleus. Notably, the Kugelkern (Kuk) gene plays a key part in this whole process, as the structure of its protein is close to that of nuclear lamins and its expression is turned up in tandem with the expansion of the nucleus [118]. Microtubule polymerization processes generate the elementary forces required for dynamics of the NE. In addition, large-scale NE deformations, which are accompanied by the development of grooves, require a buildup of microtubule polymerization into bundles coordinated via dynein. Nevertheless, microtubule bundles are unable to form grooves when the farnesylated INM protein Kuk is lacking. Kuk enhances the stiffness of the NE, while also mitigating deformations of the NE caused by the collective action of microtubule polymerization forces that are focused in bundles. The volume ratio of the cell nucleus to the cytoplasm is associated with the cell cycle [119], whereas the size of the cell nucleus correlates with the rate of RNA transcription [120]. Moreover, the size of the cell nucleus is crucial for the DNA polymerase activity [121]. Irregularities in the volume ratio of the cell nucleus to the cytoplasm are also closely linked to the appearance and progression of cancer. There is no clear universal relationship between nuclear size and cancer metastasis across all cancer types. Enhanced metastasis is associated with smaller nucleus size for small cell squamous cell carcinoma of the lung and osteosarcoma [122,123], while larger nucleus size is linked to breast, prostate, colon, and various other cancers [124,125,126,127]. This lack of consistency has prevented the development of a solid conceptual model explaining how cancer-related alterations in cell size could facilitate metastasis. However, depending on the tumor type, it continues to be used as a prognostic marker for the progression of cancer, for example, in colorectal cancer, where a unfavorable prognosis corresponds to an average expansion of the nucleus area from 3.02 to 3.42 µm^2^ [128].

### 3.2. Nuclear Volume

Why is nuclear volume so important in the progression of cancer? This is briefly explained below, as it illustrates why mechanical markers are necessary to determine or predict the progression of cancer. Cancer cells have an increased nuclear volume compared to their normal counterparts, as they often have unusually large and irregularly shaped nuclei, a condition known as nuclear hypertrophy. Therefore, the irregularity of cancer cell nuclei, including abnormal shapes, enlarged size, and volume, is a critical diagnostic characteristic. In addition, nuclear size and volume are directly linked, as nuclear size (radius) increases proportionally to nuclear volume, which in turn scales with total cell size, thereby preserving a nearly constant ratio between nuclear and cell volume [129]. This insight implies that cells can actively adjust their nuclear dimensions according to the volume of the cytoplasm, and not merely the DNA amount, to attain the most effective functioning. The increase in cell nucleus volume often leads to a larger proportion of the total cell volume taken up by the cell nucleus, which leads to a higher nuclear-to-cell volume ratio compared to healthy cells. In addition, cancer often involves ploidy alterations (DNA changes), including aneuploidy (an abnormal number of chromosomes) and polyploidy (additional sets of chromosomes), which are accompanied by larger cell nuclei. In addition, the nuclear volume alterations in cancer take place during genome duplication during the cell cycle. Notably, the size of the nucleus grows as cells undergo normal cell cycles (S/G2 phases) to replicate DNA, while cancer cells frequently disrupt this process due to unrestrained growth. Consequently, aberrant nuclear enlargement caused by replication stress represents a common feature of several cancer cell types [130]. In addition, mechanical forces that affect the tension of the cytoskeleton and the structure of the NE play a role in the development and progression of cancer and can perform important tasks; cancer cells can modify these forces to change the size of the cell nucleus (see Section 5.1). As a result, signaling pathways are activated, such as the Ras/Erk signaling cascade, which is involved in the enlargement of the cell nucleus and the number of nuclear pores and impacts trafficking into and out of the cell nucleus. The cellular microenvironment, such as extreme constriction or modified tissue elasticity, can cause variations in cell nucleus volume when cancer cells are attempting to adjust. Moreover, the physical volume of the cell nucleus affects the development and performance of nuclear structures like nucleoli and Cajal bodies (CBs) engaged in the processing of RNA, thus impacting entire transcription. Finally, the traditional characteristics of cancer cells, such as abnormalities in the size and morphology of the cell nucleus (pleomorphism), are typical indicators of malignancy that pathologists consider when determining a cancer diagnosis and evaluating its stage/aggressiveness. Essentially, the changed volume of the cell nucleus in cancer is not merely a symptomatic feature, but also mirrors the underpinning genetic instability and alterations in cell mechanics that propel the growth of the tumor.

### 3.3. Nuclear Shape

The cell nucleus usually appears spherical or ellipsoidal in form, though its internal structure is intricate and consists of multiple elements. Throughout the differentiation of cells, the expression levels of certain proteins typically vary over time, leading to considerable alterations in the shape of the nucleus. The nucleus serves as a cellular mechanical sensor, and changes in its morphology can trigger conformational alterations in chromatin inside the nucleus, which directly affect the subsequent processes of gene transcription [131,132,133]. The shape of the cell nucleus is dynamically controlled through cytoskeletal forces caused by actin/microtubule polymerization, myosin contraction, and microtubule motor activity, such as dynein and kinesin [134]. These forces can act either directly on the cell nucleus or are conveyed internally, typically through the Linker of Nucleoskeleton and Cytoskeleton (LINC) complex, which connects the nucleoskeleton (see Section 5.4) to the cytoplasmic cytoskeleton [135,136]. There is growing evidence that the cell nucleus can sense external mechanical cues and alter its morphology to adjust to different physiological and biochemical contexts. Cells attached to a soft matrix have a round nucleus [137], whereas cells attached to a stiff matrix tend to have a flat nucleus [138,139]. Nuclear deformation is clearly visible in several key life processes, including growth, development, and cellular differentiation. In what way are mechanical signals reflected in the shape of the cell nucleus? After a mechanical input is transferred to the nucleus, the reaction of the nucleus is influenced through multiple contributing factors, among them the nuclear lamina, chromatin, RNA, nuclear proteins, and several components within the nucleus, all of which are involved in its mechanical responsiveness [140]. For example, micropipette aspiration studies performed on the nuclei of HeLa cervix carcinoma cells, mouse embryonic fibroblasts and large African clawed frog eggs have demonstrated that the nuclear fiber layer serves an important protective purpose against initial deformations, while the nuclear interior mainly serves to resist extensive deformations [141,142]. The shape of the cell nucleus can be indicative of the pathological status of the cell, as abnormalities in nuclear shape have been linked to various diseases, like genetic disorders, cancer and metastatic spread, immune deficiencies, neuromuscular disorders and cardiovascular disease [143,144,145]. For this reason, investigating nuclear shape variations is crucial for gaining a deeper insight into cell functions and the emergence and evolution of diseases.

### 3.4. Nuclear Positioning

Due to its enormous size and shape-retaining properties, positioning and reshaping the cell nucleus poses a mechanical difficulty for migrating and invading cells. By transferring forces from the cytoskeleton to the nuclear outer surface, the nuclei are shaped and positioned. Force transmission can be achieved via certain connections between the NE and the cytoskeleton. The cytoskeletal forces applied to the cell nucleus can essentially cause two reactions: The cell nucleus can deform and/or undergo displacement. The forces are also capable of shifting intranuclear structures like chromatin and intranuclear structures (see Section 7). Nuclear motion arises when there is a net difference in mechanical force acting across the nucleus, whereas nuclear deformation takes place when the mechanical forces exceed the mechanical resistance of the diverse structures that constitute the nucleus. To comprehend how the cell nucleus shifts, it is essential to identify the underlying drivers and magnitudes of the competing forces that constitute the equilibrium of nuclear forces, and to appreciate how these forces dynamically evolve over the course of processes such as cell migration. During cell migratory movement, the cell needs to keep the cell nucleus in a position that preserves its polarity and deform the nucleus to squeeze through tight passageways [146]. For this reason, the nucleus is located in the rear of most migrating cells, such as cancer cells, far away from the protruding leading edge, as reviewed in [147]. The positioning and shape of the cell nucleus pose a special difficulty due to its massive size and resilience to deformation, and thus necessitate the transmission of the active forces that are generated or transmitted by the cytoskeleton toward the cell nucleus. These forces can be produced through actin or microtubule polymerization, actomyosin-mediated contraction, and/or motor activity of microtubules to cause compression, stretching, or pulling of the cell nucleus [133], and may even result in nuclear membrane rupture in several circumstances [105,106]. The cell nuclei are also asymmetrically arranged in several specialized animal tissues, like skeletal muscles, various epithelial cells, and neurons. These cases point to the idea that the positioning of cell nuclei is crucial for certain cell activities, and that abnormalities in positioning can cause malfunctions and illnesses such as cancer.

Finally, it can be said that the traditional characteristics of cancer cells like nuclear shape, nuclear positioning and nuclear size/volume and their influence on cancer development and metastasis must be fully understood before they can be used as biomarkers for cancer development or for metastatic and therefore invasive cancer cells. In the future, a thorough comprehension of the heterogeneity of tumors and the intricate, constantly fluctuating microenvironment will be critical for achieving effective, personalized, and accurate early diagnosis through the integration of these traditional biomarkers. It is therefore essential to clarify the relationship between traditional nuclear biomarkers and extracellular environmental influences, as well as the resulting mechanical properties of the cell nucleus and nuclear mechanotransduction, as outlined and discussed in the following section.

## 4. Mechanical Characteristics of the Nucleus Provide New Mechanomarkers for Cancer

The traditional properties of the cell nucleus cannot be considered independently of its mechanical properties, as they influence each other. The following section explains why the mechanical properties of the cell nucleus are relevant and which components of the cell nucleus contribute to its mechanical and traditional properties. Only recently has it been increasingly recognized that the altered mechanical properties of the cell nucleus also play a decisive role in cancer development and progression. The properties of the cell nucleus generally influence the ability of cells to move through dense ECM tissue, where it is particularly important that cells can perceive the mechanical properties, such as forces, of their extracellular environment and transmit them to the cells. This also includes transmission to the cell nucleus, enabling cells to respond directly and indirectly to changes in the mechanical environment outside the cell.

### 4.1. Why Are Mechanical Characteristics of the Nucleus Important?

The importance of the mechanical properties of the cell nucleus in cancer and its malignant progression must initially be briefly explained. In the past, at least three parameters of the cell nucleus have been identified as decisive characteristics for the development of cancer and cancer metastases, including the shape, size/volume and position of the cell nucleus, usually without establishing a connection to the mechanical properties of the cell nucleus. However, the exact universality across different types of cancer is still uncertain. Most connections between core characteristics and migration and invasion come from normal migrating cells. There is a high probability that they also apply to cancer cells. The first characteristic, the position of the cell nucleus, is widely recognized to fluctuate according to developmental stage, differentiation status, movement activity, and other physiological parameters [133]. It is therefore necessary for the cell nucleus to identify and transmit mechanical signals so that it can respond to changes in its position and mechanical characteristics. The second characteristic of the nucleus is that its shape is highly dynamic and can be impacted by both cytoplasmic and nucleoplasmic elements. Microtubules and the actin cytoskeleton have both been proven to impact the determination of nuclear shape [148]. There is also evidence that actomyosin-dependent contractility is implicated in the rotational movement of the cell nucleus [149]. Inside the nucleoplasm, lamins are key to shaping the nucleus by working from the inside out, where lamin A/C is a major factor in controlling the stiffness of the nucleus [145,150]. How can these nucleoskeletal filaments impact the shape of the nucleus? The actin cytoskeleton can act in two ways. First, it can form a perinuclear cap encircling the cell nucleus. The perinuclear cap is a network of actin filaments that wraps around the nucleus in a cap-like structure and directly affects the morphology and positioning of the nucleus. Second, the perinuclear actin cap forms a special “dome” of actomyosin fibers (actin stress fibers) that enclose and constrain the cell nucleus in the apical direction, thereby sustaining its flattened, discoid shape in spreading cells. Through this process, actin and actomyosin contractility transfer mechanical forces from the outside of the cell via focal adhesions to the cell nucleus and influence its morphology by evoking invaginations and wrinkles of the nucleus. Moreover, rearrangement of the nucleus in the course of cell migratory movement or differentiation is caused by actin-driven forces that are critical for the deformation of the cell nucleus. Apart from the actin cytoskeleton microtubules of the cytoskeleton contribute to nuclear mechanics. They offer structural support, as microtubules can provide compressive resistance. Microtubules are the stiffest elements of the cytoskeleton and can provide structural stiffness to the cell because they can withstand large compressive forces from the surrounding contractile actin filament scaffold, thereby avoiding uncontrolled and severe deformation of the cell nucleus. In addition, microtubules can control the actomyosin-induced inward folds, thereby governing the amount of nuclear folding. Microtubules can play a role in active deformation. For example, in certain cells such as myeloid progenitor cells, microtubules bundle together to envelop the cell nucleus and are actively involved in the formation of large invaginations that are crucial for lineage-specific expression of genes. Microtubules facilitate the positioning of the cell nucleus by pushing and pulling it. Together with motor proteins such as dynein and kinesin, microtubules exert forces to rotate and position the cell nucleus throughout cell motility and development. As stated before, coordination and interaction between the cytoskeleton and the elements of the cell nucleus is ensured by the integration of the LINC complex, which consists of nesprin proteins on the ONM and SUN proteins on the INM. Nesprins attach directly to actin or microtubules, which occurs typically through motor proteins. SUN proteins attach to the nuclear lamina via direct interaction with lamin A/C, maintaining internal structural stability and governing nuclear stiffness. There exists a dynamic interplay between the cooperating networks. For instance, a perturbation of actin can cause microtubules to accumulate in the perinuclear region and lead to invaginations. Consequently, a breakdown in this mechanical connection can result in nuclear morphological abnormalities, which are linked to diseases like cancer. In addition, changes in nuclear mechanics, such as those caused by chromatin organization, can lead to inside-out signal transduction, which in turn can remodel the cytoskeleton and thereby induce a feedback loop.

Third, it is characteristic of numerous types of cancer that there are alterations in the size of the nucleus when the cancer cells become metastatic. For instance, elevated metastasis is linked to decreased nuclear size in small cell squamous cell carcinoma of the lung and osteosarcoma [122,123] whereas it is associated with increased nuclear size in several other cancers, including breast, prostate, and colorectal cancers [124,125,126,151]. Moreover, in colorectal cancer, a worse prognosis is associated with an average rise in the nuclear surface area from 3.02 to 3.42 µm^2^ [128]. The idea that nuclear size could be a factor in metastasis was initially dismissed because, according to the type of cancer, both an enlargement and a reduction in nuclear size could be associated with a higher rate of metastasis [152]. Nevertheless, recent investigations into nuclear mechanics and the interconnectivity between chromatin, the nucleoskeleton, and the cytoskeleton suggest that alterations in this interconnectedness may have substantial consequences for cellular motility and invasiveness [153]. In this context, another investigation has critically noted that the reversal of tumor-type-dependent alterations in nuclear size relates to decreased cell motility and invasiveness [152]. Finally, it can be concluded that due to the weaknesses of current biomarkers for cancer cells or even metastatic cancer cells it is necessary to identify other markers for cancer cells, such as mechanomarkers like softness/stiffness, traction forces or viscoelasticity. Since certain mechanomarkers, such as softness/stiffness, are also critically discussed despite already being in use [154], the search for reliable mechanomarkers is by no means complete, but rather in full swing.

### 4.2. How Contribute Nuclear Elements to Mechanical Characteristics of the Cell Nucleus?

In terms of materials science, the cell nucleus can be regarded as a multiscale material, as it comprises diverse structures of vastly different sizes [155]. The cell nucleus contains small nucleoproteins and RNA molecules measuring only a few nanometers in size, nucleoli and micrometer-sized nucleoli harboring hundreds of diverse proteins and RNA molecules in large quantities, as well as long chromosomal molecules covering micrometer-sized spaces [156,157,158]. Within the cell nucleus, chromosomes form a porous framework that encloses the voids between chromatin [157,159,160,161]. This complex organizational scaffold imparts scaling-dependent structural properties, rheological characteristics, and mechanical traits to the cell nucleus. Among the key mechanical cues of the nucleus are stiffness and elasticity. Specifically, the cell nucleus is considerably stiffer than the encompassing cytoplasm, withstanding deformation and functioning as a shock absorbing mechanism, whereby the stiffness is contingent upon the nuclear lamina and chromatin. Another mechanical property of the cell nucleus is the viscoelasticity. It possesses both solid-like (elastic) and liquid-like (viscous) attributes, enabling both structural robustness and intracellular rearrangement. The nucleus exhibits scale-dependence, as its characteristics rely on the scale. For example, small motions are associated with chromatin pores filled with fluid that are associated with small deformations of the nucleus, whereas larger deformations affect complete chromosomes or whole nucleoli. Several structural elements of the nucleus resemble its mechanical characteristics. First, the NE is a double-layered membrane that is critical for maintaining overall structural integrity of the cell nucleus. Second, the nuclear lamina, which is a more stable viscoelastic scaffold composed of intermediate filaments, such as lamins, that serve as important structural anchors and establish connections to the cytoskeleton. Third, there is chromatin, which constitutes a dense and porous framework that occupies the majority of the nuclear space. Chromatin plays a major part in its mechanical strength and flexibility of the cell nucleus [162]. Chromatin, which consists primarily of DNA and histone proteins, exists in nucleosomes and is additionally packed into euchromatin (loosely compacted) and heterochromatin (tightly compacted) [163]. There are three defined chromatin states [164]. There is a spectrum of DNA accessibility states inside the cell nucleus, ranging from hyperaccessible states, commonly referred to as “open” chromatin, to more moderate accessibility states referred to as “permissive” chromatin, to less accessible or repressive states referred to as “closed” chromatin [165]. Open and permissive states are frequently transcriptional active chromatin and commonly referred to as euchromatin, while closed states are generally termed heterochromatin. The organized chromatin compartments occupy specific positions within the nuclear periphery, and beneath the nuclear lamina of the majority of mammalian cells lies a dense heterochromatin layer [166,167]. These heterochromatin regions associated with the lamina are marked with low gene density, low transcriptional activity, and an increased level of repressive modifications of histones, such as histone 3 lysine 9 dimetylation (H3K9me2), histone 3 lysine 9 trimethylation (H3K9me3), and H3K27me3 [167]. Accordingly, impairment of methyltransferases, which place H3K9me2 (*Ehmt2*/*G9a*) and H3K9me3 (*Setdb1*, *Suv39H1*, and *Suv39H2*), interferes with gene silencing and chromatin placement at the lamina [163,168,169]. The biochemical basis of heterochromatin assembly is based on gene silencing in conjunction with methylation of histone tails like H3K9me3, which in turn facilitates the attachment of heterochromatins protein 1 (HP1) [170,171,172,173]. Once bound, HP1 proteins then dimerize and connect neighboring chromatin fibers to each other [174,175,176,177]. It is noteworthy that HP1 proteins experience liquid–liquid phase separation from the nucleoplasm and create liquid condensates that wrap around the heterochromatin [178,179]. This means that the genome in the cell nucleus is organized and packaged in a specific way to effectively suppress gene expression. In addition to the distinct impacts of HP1α-mediated phase separation and loop extruders, chromatin bending stiffness may add to chromosomal architecture through interplay with the NE and topology-based restrictions [180].

Forces originating from the external microenvironment can deform and rearrange chromatin to alter overall gene expression profiles [181]. How is mechanical stress broken down in the cell nucleus? How does chromatin cope with mechanical stress? How is chromatin safeguarded from mechanical stress? Chromatin changes its mechanical condition in accordance with deformations to preserve the integrity of the genome [109]. While mechanical stress buildup in tissues can disrupt the integrity of the tissue [182,183], epithelial layers can withstand severe deformation and mechanical stress with almost no apparent evidence of damage [184,185]. The integrity of epithelia is governed in a tension-independent fashion through the connection of tissue stretching and surrounding matrix hydraulics [183]. It is therefore plausible that non-cancerous epithelial cells, like skin epidermal stem/progenitor cells (EPCs), which are subject to strong dynamic mechanical forces in their natural surroundings [186,187], have built up strong ways to protect their genome from mechanical stress. This stands in stark opposition to cancer cells, in where mechanical deformations result in nuclear ruptures and damage to DNA [105,106,188]. Stretching induces immediate nuclear deformation, causing piezo1-driven calcium liberation of the ER, which diminishes the lamina-associated H3K9me3 heterochromatin and subsequently softens the nucleus [109]. Blocking calcium inflow or raising heterochromatin amounts by forcing the expression of the H3K9me3 methyltransferase Suv39H1 prevents chromatin mechanics from being changed, which ends up harming the DNA [109]. Exposure to high levels of stretching over a long period of time causes the monolayer to align perpendicular to the stretch in a manner unrelated to the deformation route of the cell nucleus and propelled by cell–cell adhesion [109]. This multicellular structural organization is necessary to disperse mechanical stress away from the cell nucleus, so that cells can reestablish their stationary nuclear and chromatin framework for sustained mechanical protection. It can therefore be assumed that nuclear deformation has an important function in essential cellular processes, among them muscle contraction, cell motility, and the disease pathogenesis in humans [189]. Fourth, the LINC complex couples the nuclear lamina and the cytoplasmic cytoskeleton, enabling the transfer of forces [190]. Fifth, the nucleoskeleton, which can be regarded as the nuclear matrix and constitutes an internal nuclear framework consisting of lamin and chromatin, which is integral to the structure of the cell nucleus. The nuclear matrix provides a protein-based scaffold that supplies structural stability and positional arrangement for nuclear processes [191,192].

Since it has been established that the tension of the nuclear membrane tension correlates with the amount of lamin A [193], the question arises whether the impact of stretching on H3K9me3 correlates with lamin A. Two types of stem cells, EPCs and mesenchymal stem cells (MSCs), exhibited high lamin A concentrations and high nuclear elastic moduli and reacted to 40% stretching with a decrease in H3K9me3 amounts [109]. Conversely, the epithelial cancer cell line SCC9 and fibrosarcoma cell line HT-1080 displayed low lamin A concentrations and low nuclear elastic moduli and revealed a slight rise in H3K9me3 concentrations under stretching [109], aligning with earlier findings [108]. Notably, elevated lamin-A expression in HT-1080 cells sensitized them to stretch-induced intracellular Ca^2+^ liberation, whereupon these cells also reacted to stretching with reduced H3K9me3 concentrations [109]. In contrast, the reduction of lamin A in EPCs with small interfering RNA (siRNA) led to a sharp decrease in the elastic modulus of the cell nucleus and protected against a further reduction of H3K9me3 levels and stiffness of the cell nucleus [109]. Overall, these outcomes point to the fact that the cell nucleus and the connected endoplasmic reticulum (ER) membranes perceive deformations, whose sensitivity is controlled through the stiffness of the cell nucleus/membrane tension under equilibrium conditions. Cell/nucleus deformation of cells with tight nuclei and elevated membrane tension induces the liberation of Ca^2+^ from the ER, which promotes actin polymerization around the nucleus and heterochromatin rearrangements to enhance chromatin movement and reduce perceived NE tension. These alterations probably ease mechanical energy dissipation to avoid DNA injuries. Based on these findings, it has been demonstrated that nuclear stiffness is a key mechanical characteristic of the cell nucleus, which is mostly controlled by the nuclear lamina, chromatin, nuclear pore complex (NPC), and other elements [194,195]. A key factor in the nuclear structures, the expression rate and mode of assembly of lamin A control the stiffness of the cell nucleus. The deficiency of lamin A results in reduced nuclear stiffness, thereby elevating cellular sensitivity to mechanical stress and disrupting critical physiological and biochemical activities, including mechanotransduction signaling, wound repair, cell growth, and cell fate determination [196,197,198]. Type B lamins also assist in preserving the stiffness of the cell nucleus [199,200]. The lack of any type of lamins has a considerable effect on the stiffness of the cell nucleus. Epigenetic alterations and chromatin conformations are also implicated in controlling the nuclear stiffness. The application of histone deacetylase inhibitory agents to elevate the amount of euchromatin and the application of histone methyltransferase inhibitory agents to reduce the amount of heterochromatin are both capable of reducing the stiffness of the cell nucleus [201,202]. Moreover, the transcription factor PBX-regulating protein-1 (PREP1; synonymously referred to as PKNOX1) impacts nuclear stiffness through modifying the constitution of the nuclear envelope and the expression of the INM proteins SUN1, SUN2, and lamina-associated polypeptide 2 (LAP2), consequently influencing the interplay and mechanical force transmission between the nucleoskeleton and cytoskeleton [8].

The nucleus’s stiffness is related to its functional role, whereby nuclei from higher-density tissues like muscle and bone have greater stiffness compared to those from softer tissues such as adipose tissue or neural tissue [203]. An abnormal stiffness is a sign of certain disorders. In Hutchinson-Gilford progeria syndrome, *LMNA* gene mutations lead to aberrant lamin A protein processing, resulting in its excessive deposition on the NE, elevated nuclear stiffness, alterations in chromatin structure, accelerated cellular aging, and impaired stem cell homeostasis or differentiation [204,205]. In contrast, decreased nuclear stiffness (elevated softness) may point to enhanced fluidity of cancer cells [206,207] and may possibly act as a mechanomarker for increased metastatic capacity, as reviewed in [154]. The nuclear stiffness and fluidity of highly, intermediate, and non-metastatic immortalized prostate cancer cell nuclei and normal prostate epithelial cell line nuclei have been examined. While fluidity cannot be relied upon to differentiate between cancers with varied metastatic capacities, stiffness can be applied in most situations [208]. A significant difference in stiffness was also observed between nuclei of highly metastatic prostate cancer and nuclei of mildly metastatic prostate cancer and normal prostate cells [208]. The stiffness of the cell nucleus is experimentally quantifiable, for instance by exploiting nuclear creep experiments using a microfluidic channel with a narrow constriction. For example, the nuclear stiffness of highly metastatic prostate cancer cells PC-3 was around 0.4 kPa, whereas, moderately metastatic prostate cancer cells DU-145 displayed a nuclear stiffness of around 4 kPa and normal prostate cells RWPE1 possessed a nuclear stiffness of around 6.5 kPa [208]. In primary human endothelial cells, whose stiffness was measured using atomic force microscopy (AFM), the nuclear areas of intact cells (5.59 ± 1.55 kPa) exhibited a relatively higher average elastic modulus compared to the non-nuclear areas (1.47 ± 0.77 kPa) and the nuclei exposed in situ (22.06 ± 7.29 kPa) were considerably stiffer compared to the nuclear areas of intact cells [209]. In general, the nuclear stiffness seems to be cell type specific.

On the one hand, this seems to be due to the fact that the stiffness of the cell nucleus is affected by several variables, among them cell type, cell condition, stage of the cell cycle, extracellular matrix stiffness, and the mechanical milieu within the cell. On the other hand, nuclear stiffness can be a dynamic mechanical characteristic with values obtained through experiments, although they require explanations based on particular biological contexts and experimental requirements. Most biophysical techniques that are predominantly used to measure nuclear stiffness, such as atomic force microscopy, micropipette aspiration, substrate strain, microfluidic constriction, and particle tracking microscopy, determine nuclear stiffness at a specific point in time, whereby repeat measurements of the same cell nucleus are directly possible, but they do not allow the dynamic course of cell nucleus stiffness to be determined [162,210,211,212]. Notably, another weakness of the measurements is that the environment during the measurement is very artificial and the measurements predominantly do not take place in a 3D environment (or cannot take place). Recently, there has been an increase in measurements of nuclear stiffness that take dynamic stiffness measurements into account, for example, in serial microfluidic constriction setups, image elastography based on displacement field analysis of fluorescently labeled core elements such as histones, and Brillouin-Raman micro spectroscopy, which uses frequency-shifted scattered light to derive the local elasticity and viscosity of biological tissue, resulting in better internal comparability of measured results [210,212,213]. Consequently, analysis of nuclear viscoelasticity may be more reliable [214,215].

In summary, the response of the cell nucleus to cytoskeletal forces is governed by the mechanical characteristics of the structures inside the cell nucleus, which comprise the nuclear lamina, chromatin, nuclear matrix including the nucleoskeleton, nucleoli, RNA, and proteins. The following section explains in depth the contribution of various nuclear structural components, such as peripheral nuclear elements and central elements to the mechanical behavior of the cell nucleus in response to mechanical forces, along with the sources of cellular forces acting on the cell nucleus.

### 4.3. Impact of Small Molecules and Inhibitors on Nuclear Mechanics and Mechanotransduction

Small organic molecules and chemical inhibitory agents are indispensable instruments for investigating nuclear mechanics and mechanotransduction, as they can be used to influence the structure of the NE, the connection between the nucleus and the cytoskeleton, and organization of chromatin (Table 1). These compounds impact the stiffness and morphology of the cell nucleus, as well as its capacity to perceive and react to extrinsic mechanical forces. There are four major groups of inhibitory substances: first, there are the inhibitors of nuclear structure and lamina that impact nuclear mechanics. These compounds are directly affecting the structural elements of the cell nucleus, especially lamin A/C, which is responsible for the stiffness and shape of the cell nucleus. For instance, betulinic acid enhances the breakdown of lamin A/C, resulting in softening of the cell nucleus and enhanced migration within confined environments. Another example includes farnesyltransferase inhibitors such as lonafarnib, which are utilized to inhibit farnesylation of progerin in laminopathies. They decrease the abnormal stiffening of the NE and the nuclear blebbing induced through progerin buildup. Moreover, histone methyltransferase inhibitors like chaetocin, which impairs SUV39H1 that decreases H3K9me3 levels, results in reduced heterochromatin amounts, decreased nuclear stiffness, and elevated nuclear blebbing/rupture. BIX01294 impairs G9a, thereby diminishing H3K9me2, which leads to enhanced stiffness of the cell nucleus and decreases blebbing, thereby enhancing the condensation of specific chromocenters [216]. Transcription inhibitors, such as actinomycin D, inhibit the formation of nuclear blebbing and fragmentation by disrupting the motor activity of RNA polymerase II, which can otherwise lead to deformation of the nuclear periphery regardless of nuclear stiffness. To quantify irregularity in the nucleus in an unbiased fashion, an automated elliptical Fourier analysis technique was designed and implemented to measure the elliptical Fourier coefficient (EFC) ratio [217]. The EFC ratio measures the irregularity of the contour of the nucleus; lower EFC values correspond to a high degree of irregularity. Treatment of HT1080 human fibrosarcoma cells or mouse embryonic fibroblasts with HDAC inhibitors resulted in nuclear blebbing as a result of chromatin decompaction and nuclear softening [218]. In line with these results, the two compounds that inhibited HDAC, such as salermid (SIRT = class III inhibitor) and panobinostat (pan-class I/II inhibitor), enhanced the irregularity of the cell nucleus.

Second, there are the inhibitors of nuclear-cytoskeletal coupling that target the LINC complex. Metalloproteinase (MMP) inhibitors influence ECM-dependent signal transduction. The inhibition can result in a decrease in nuclear stiffness through indirect effects on the phosphorylation of lamin A/C, thereby impairing the cell’s capacity to convey force from the ECM to the nucleus. Microtubule disruptors such as nocodazole cause microtubules to depolymerize and interfere with the force transmission necessary for positioning the nucleus and sustaining its shape. Taxanes like paclitaxel stabilize microtubules and lead to irregular cell nucleus shape and micronucleation.

Third, there are inhibitors of force generation and transmission that specifically act on the actomyosin cytoskeleton. These compounds regulate the cellular tension (pre-stress) produced by the cytoskeleton, which is subsequently conveyed to the cell nucleus. For example, myosin II inhibitors such as blebbistatin decrease the contractility of actomyosin, lower the forces acting on the cell nucleus, cause softening of the cell nucleus, and reduce the nuclear phosphorylation of mechanosensitive proteins. Another example are Rho kinase (ROCK) inhibitors such as Y-27632, which impair RhoA-dependent contractility, thereby reducing cytoskeletal tension and thus diminishing the mechanical cues acting on the NE.

Fourth, there are inhibitors of nuclear transport that impact mechanotransduction processes. Small molecules can interfere with the transport of mechanosensitive transcription factors, such as YAP/TAZ, into the cell nucleus, which is an important part of mechanosignaling. These include selective inhibitors of nuclear export (SINE) such as selinexor/KPT-330, which bind covalently to CRM1/XPO1 and block the export of cargo molecules from the cell nucleus. Another classic inhibitor is leptomycin B, which impairs CRM1-driven nuclear export.

### 4.4. Linkage of Nuclear Mechanics/Mechanotransduction and Cancer Cell Migration and Invasion

The cell nucleus represents an obstacle to non-proteolytic cell migration in intricate tissue microenvironments [146]. These crowded surroundings are frequently burdened with a densely packed ECM and cell assemblies, creating obstacles that demand the cells to squeeze their nuclei into tight spaces and utilize the nucleus as a mechanical measuring device to recognize and move toward the pathway of least resistance [219]. Incorrect positioning of the cell nucleus or its decreased deformability as a result of factors such as increased nuclear stiffness [220,221,222], decreased cortical contractility [223,224] disturbances in the mechanism of force transmission between the cell nucleus and the cytoskeleton [225], have been implicated in impaired 3D migration and invasion. Beyond the functions involved in cell migration discussed above, the cell nucleus has been proposed as a central feature in a special cell migration mechanism referred to as the nuclear piston mechanism [226]. This process is marked by the development of blunt, cylindrical, pressure-driven protrusions referred to as lobopodia and by unpolarized cell signals such as PIP3, Rac1, and Cdc42 [227]. Based on this process, the cell nucleus physically separates the cell into a front and rear section, with forces generated by actomyosin driving the cell nucleus forward and thus generating pressure in the front section of the cytoplasm [226], with the cell nucleus acting similarly to a piston in this context. In accordance with this model, hydrostatic pressure measurements utilizing the servo-zero method on primary human fibroblasts that migrated in a cell-derived 3D matrix demonstrated uneven hydrostatic pressure across the cytoplasm, with anterior values of approximately 2400 Pa and posterior values of approximately 900 Pa [226]. This gradient of pressure throughout the cell may arise from mechanical compression of the front compartment as a result of the piston-like cell nucleus itself, from internal osmotic pressure differences, or from differences in actomyosin forces across the cell. Regardless of the origin of this gradient, slow hydrostatic pressure adjustment across the cytoplasm can be anticipated because of the high resistance to water flow provided by the soft and porous cytoplasmic architecture [228].

Several studies have found increased cortical contractility in the posterior part of the cell, for example, in interneurons [229,230,231,232], indicating that increased hydrostatic pressure in the posterior compartment, in contrast to experimental measurements of hydrostatic pressure, presses the nucleus forward, thus supporting its forward propulsion through 3D narrowings. Is this elevated hydrostatic pressure behind the cell nucleus actually sufficient to push the cell nucleus forward, similar to the pressure-driven movement seen in amoeboid cells during cytoplasmic streaming, or is it merely causing a local enlargement of the cell nucleus? [229]. Conversely, the forward movement of the cell nucleus can be facilitated by actomyosin traction forces at the leading edge, which are supported by proteins of the nucleoskeletal cytoskeletal network [223,226], specifically myosin IIA, vimentin, tropomyosin (Tpm 1.6/7), and nesprin-2, all of which are enriched at the anterior region of the cell nucleus [223,226,233]. This caused the cell nucleus to travel forward regardless of the rear edge of the cell. This caused the cell nucleus to travel forward regardless of the rear edge of the cell. Whereas myosin II activity inhibition at the back of the cell was ineffective, myosin II activity inhibition in front of the cell nucleus impeded the nucleus’s forward motion, reduced hydrostatic pressure at the leading edge, and led to retraction of the leading edge [226]. All of this promotes a mechanism in which forward-directed cytoskeletal forces propel the motion of the cell nucleus.

How can these high-pressure protrusions promote the expansion of the protrusions and the successful migration of lobopodial cells in intricate 3D settings, especially since high hydrostatic pressure at the leading edge of the cell would cause a temporary loss of small volumes of water from the protrusion? This water leakage would then result in the protrusions shrinking, contrary to the results of the experiments. The forward movement of the cell nucleus could increase the local hydrostatic pressure at the leading edge, which would then cause the plasma membrane to stretch, causing the opening of mechanosensitive ion channels. This causes ions to inflow into the protrusion, whereby osmotic pressure dominates hydrostatic pressure, promoting the extension of the protrusion and facilitating rapid lobopodial motility [230]. The proposed mechanism by which increased hydrostatic pressure in the protrusion opens mechanosensitive ion channels has not been explored. An initial increase in hydrostatic pressure within the protrusion would lead to a shrinkage of the cell protrusion, which may lower the plasma membrane tension.

An alternative mechanism could be that the cell nucleus advances like a piston, thereby accumulating osmolytes at the leading edge of the cell and thereby inducing expansion of the protrusion through osmotic pressure. This initial enlargement can drive the activation of mechanosensitive channels [230], which additionally raise the local osmotic pressure, leading to a fast enlargement of the protrusions and rapid cell migration. The concept of osmotic pressure as the main driver for the expansion of protrusions in lobopodial cells has not yet been comprehensively investigated, but it is biophysically reasonable and may represent a potential avenue for future research. In summary, these results demonstrate the importance of nuclear mechanics and mechanotransduction for cancer cell migration and invasion. Cancer cells without the ability to deform their cell nucleus appear unable to migrate through narrow spaces unless they can overcome the obstacle by enzymatic degradation.

## 5. Peripheral/Interfacial Nuclear Mechanosensory Elements Regulate Mechanosensing and Mechanotransduction

**Figure 3:**
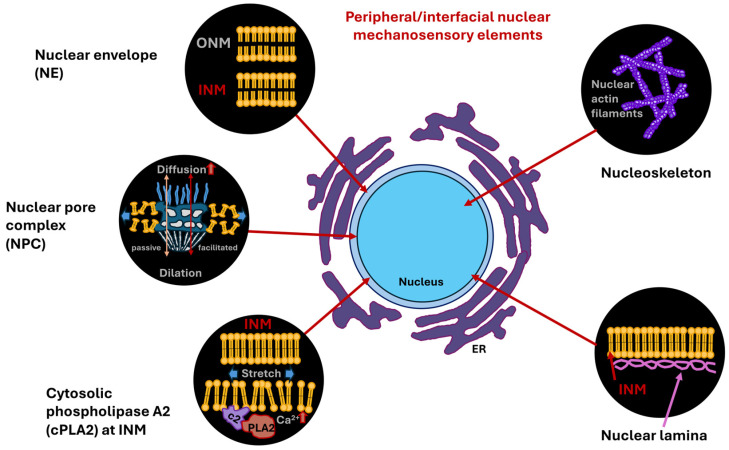
Mechanotransduction of the cell nucleus is regulated through a broad variety of peripheral/interfacial nuclear elements, such as the NE, which is composed of an ONM and an INM. The NE harbors the NPC that can be dilated in tensioned NE, whereby the passive diffusion and the facilitated diffusion are elevated. Moreover, the NE can incorporate the cytosolic phospholipase A2 into the INM upon mechanical stretching and intranuclear Ca^2+^ ion level increase. The nucleus comprises a nucleoskeleton that is composed of nuclear actin filaments. Underneath the INM lies the nuclear lamina.

The peripheral nuclear mechanosensory elements that perform mechanosensing and mechanostransdcution contribute to or interact with the nuclear membrane. Moreover, they are critical in forming the interface between the nucleus and the cytoskeleton. From a traditional viewpoint, the cell nucleus can be regarded as a multiscale viscoelastic material consisting of at least seven main elements with differing characteristics: the NE, the nuclear lamina, the nucloeskeleton, the nuclear bodies, the nucleoplasmic liquid and the chromatin scaffold [234]. In addition, some of the main nuclear (mechanosensory) elements can be subdivided, such as the nuclear membrane that compromises the NPCs and the INM-recruited cytosolic phospholipase A2 (cPLA2). Moreover, the group of nuclear bodies acting as mechanosensors, contain the nucleolus, the Promyelocytic leukemia nuclear bodies (PML-NBs), the CBs, including Gemini of Cajal bodies (GEMs), and the nuclear speckles. The following section explains the main components of the cell nucleus in terms of their function as mechanosensory and mechanotransduction elements (Figure 3). Before the major components are discussed, it needs to be said there are similarities and differences of the mechanosensing at the plasma membrane and the NE. Together with the plasma membrane, the NE constitutes the biggest sensory interface of a cell. They both identify mechanical deformations, but work in different locations and contexts. Through its direct contact with the extracellular environment, the plasma membrane can detect small, localized deformations throughout the whole cell surface. In sharp contrast, the NE, which is strengthened by a shock-absorbing lamina and isolated from the cell surface through the cytoplasm and cytoskeleton, has a better ability to perceive major deformations of the cellular body.

The incorporation of these overlapping but non-redundant signals permits the cells to separate peripheral from central and benign from potentially lethal mechanical cues, consequently governing cellular motility, proliferative capacity, and differentiation.

### 5.1. Nuclear Envelope (NE)

The NE consists of the INM and outer nuclear membrane (ONM), which are connected to one another and to the endoplasmic reticulum. Interaction between the nucleoplasm and cytoplasm is critically important for the functioning of the cell [235]. Three independent mechanisms exist for transmitting signals over the NE. First, using a conventional mechanism small proteins and molecules are able to diffuse across nuclear pores, whereas larger assemblies, such as ribosomal subunits, are selectively conveyed across nuclear pores [236]. Second, as an alternative possibility, various complexes, like some virus envelopes, exit the inner nuclear membrane through the process of nuclear blebbing, thereby they can diffuse in membrane-bound subcompartments through the perinuclear region and subsequently merge with the outer nuclear membrane [237]. The third way is that mechanical forces can be transmitted as a mechanical mechanism directly over the NE via conserved physical connections, namely the LINC complexes. Mechanical force transmission through the NE is necessary for the correct placement of the cell nucleus and the dynamics of chromosomes during meiosis [238,239]. The double membrane contains NPCs that vary due to nuclear plasticity. The two membranes connect at the NPCs, which enable transportation between the cell nucleus and the cytoplasm. In addition to isolating the genome region from the cytoplasm, the NE offers mechanical reinforcement to the cell nucleus via its lamina [144], which is a meshwork of lamin and lamin-binding proteins that lies beneath the INM. The lamina clamps itself to the INM and also to peripheral DNA and consequently chromatin. A-type lamin (Lamin A/C) and B-type lamin (Lamin B1/B2) proteins constitute the most important intermediate filament proteins that comprise the structural framework of the nuclear lamina. They are necessary for the shape of the nucleus, its mechanical stability, the arrangement of chromatin, and the regulation of genes, but they differ in their specific functions and their assembly mechanisms [240]. B-type lamins (B1/B2) are essential for all cells and ensure elasticity as it is needed in cell migration, whereas A-type lamins confer additional stiffness to differentiated cells, whereby they stiffen the cell nucleus by reducing its deformability. The LINC complex attaches cytoskeletal components, including actin microfilaments, microtubules, and intermediate filaments, to lamins, chromatin, NPCs, and additional nuclear membrane proteins [241,242]. This connection constitutes a mechanical link between the nucleoskeleton and the cell cortex, permitting the transmission of mechanical cues from the cell surface to the nucleus, ultimately leading to their propagation to chromatin and nuclear membranes [243].

**Figure 4:**
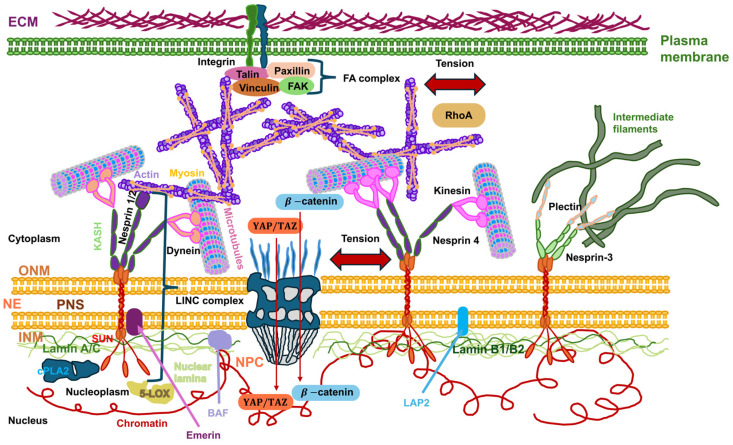
The characteristic features of the LINC complex are provided in a schematic drawing. The INM and the ONM together form the NE, which encloses a perinuclear space (PNS). NPCs span the INM and ONM and enable the exchange of macromolecules between the cell nucleus and the cytoplasm and vice versa. The LINC complex couples the NE directly with the cytoskeleton, consists of SUN and Nesprin proteins and is coupled to the INM and ONM. SUN1 and SUN2 reside in the INM and engage with nesprins, which are situated in the ONM, inside the PNS. The KASH domains of nesprins interface with cytoskeletal components in the cytoplasm. Nesprin-1 and Nesprin-2 tether microtubules with filamentous actin (F-actin), while Nesprin-3 connects to IFs through plectin and Nesprin-4 engages with microtubules through kinesin. Nesprins are tethered to the NE through the engagement of their C-terminal short Klarsicht, ANC-1, and syne homology (KASH) domain with the C-terminal region of SUN proteins in the PNS, and are thus indirectly connected to the nuclear lamina, NPC, and chromatin. Nesprin-1, Nesprin-2, and Nesprin-4 can bind to microtubules through interacting with kinesin and dynein, whereas Nesprin-3 is physically linked to the intermediate filament vimentin through the plectin protein. SUN1 and SUN2 interface with lamins type A/C (LA/LC) and lamins type B (LB1/LB2) as well as emerin. Extracellular mechanical signals are detected through the ECM and transferred across the plasma membrane via integrins, which attach the ECM to FA complexes. These FA complexes are linked to the actin cytoskeleton and transform mechanical cues into biochemical cues via FAK-facilitated phosphorylation. Thus activated FAK phosphorylates other FA proteins like paxillin, and enlists other kinases like Src kinase that triggers a cascade of signals that guide cell functions, such as MAPK/ERK that impacts cell growth and differentiation, and RhoA/ROCK, which regulates cytoskeletal tension via ROCK impairment and subsequently promotes cell motility by phosphorylating key proteins such as Raf and ROCK, respectively. Intrinsic mechanical forces are conveyed via the actin cytoskeleton and trigger a RhoA-facilitated reorganization of the actin cytoskeleton to promote cell adhesion and migration. Through shear stress, molecules like β-catenin and YAP/ TAZ are conveyed via the cytoskeleton through the LINC complex to the cell nucleus, enabling nuclear mechanical transduction and subsequently the activation of the transcription of mechanosensitive genes.

Both the NPCs and the nuclear lamina function as a physical barrier separating the nucleoplasm from the cytoplasm, thereby preventing cytoplasmic constituents from entering the nucleus and creating a distinct space for the synthesis and processing of DNA and RNA [244]. The deterioration of NE integrity and the selectivity of nuclear pores is a normal part of aging and is a hallmark of a whole bunch of human illnesses, like cancer [245]. Apart from the NPCs, another complex is critical for its function, such as the LINC complex (Figure 4). The LINC complex is essential in transducing mechanical signals from the environment into the cell nucleus and vice versa. It serves as a physical connection over the nuclear envelope, connecting the internal framework of the cell (cytoskeleton) to the interior of the nucleus, enabling the nucleus to perceive and react to external mechanical forces such as tension, shear force, or substrate stiffness, impacting gene expression, nuclear morphology, and cell behavior. It plays a role in aging and microgravity [246]. SUN proteins like Sad1/UNC-84 are critical elements situated in the INM of the NE, where they interact with KASH proteins in the outer nuclear membrane to form connectors, the LINC complexes, that join the nuclear skeleton (lamins) to the cytoskeleton, enabling nuclear localization and transmission of force. Their C-terminal SUN domain protrudes into the perinuclear lumen to engage with KASH proteins, thereby connecting the nucleus to the structural network of the cell [247]. The Nesprin-2, which contains the c-terminal KASH domain can undergo force dependent dynamic remodeling [247].

KASH proteins like Klarsicht/ANC-1/Syne homologs are present in the ONM of the NE, creating important linkages between the cell nucleus and the cytoskeleton. Together with their partners in the INM, the SUN proteins, they form LINC complexes that convey forces and anchor the cell nucleus at its place (Figure 4). Beyond the important LINC complex, there is the nuclear membrane protein emerin. Emerin works together with other nuclear membrane proteins to regulate the organization of chromatin, cell signaling processes, and gene expression. For chromatin organization at the INM, emerin interfaces with both HDAC3 and histone methyltransferases (HMTs), where it is kept in place, at least in part, by directly attaching to lamin A [248] based on the diffusion-retention model [249]. The lamin A binding domain and the transmembrane domain of emerin are both needed so that it can be kept in the INM [250]. Emerin fulfills key roles in mechanotransduction and nuclear shape adjustment, lines up into nanometer-sized domains on the INM. The size and emerin coverage of these nanodomains vary with mechanical stress and emerin mutations linked to Emery-Dreifuss muscular dystrophy (EDMD) [251]. Additionally, a straightforward reaction-diffusion model clarifies the self-organization of emerin nanodomains [251]. Apart from the LINC complex and emerin, mechanotransduction is also influenced by the NPC. The mechanoregulation can occur on several time scales, such as fast via the NPC or slow via splicing of, e.g., vinculin or fibronectin. There are two main mechanisms of nuclear mechanotransduction: the dilation of nuclear pores and the cPLA2 signaling cascade [252].

### 5.2. Nuclear Pore Complex (NPC)

In general, NPCs adapt their diameter in response to membrane tension, which emphasizes the relevance of investigating the effect of changes in pore size on molecular trafficking across the NPC [253,254]. In line with these findings, it has been reported that pulling forces exerted by the nuclear membranes cause NPCs to stretch and increase in diameter, while releasing these forces results in NPCs narrowing. Therefore, regulation of the size and morphology of the nucleus is intrinsically connected to the conformational properties of NPCs and the activity of the nucleocytoplasmic trafficking system [255]. Latest breakthroughs in understanding the exact protein structure of the NPC confirm its dynamic capacity to modify pore size through expansion and contraction [256,257,258,259,260]. There is a correlation between pore size and transport rate, implying that minimal adjustments of the pore size can dramatically enhance the molecular flow rate over several magnitude orders [261]. This finding underscores the decisive influence of pore size on molecule transportation and emphasizes the importance of governing the NPC’s architecture for proper functioning of the cell.

#### 5.2.1. Nucleoporins (Nups)

There are about 30 distinct genes in eukaryotes codifying for NPC constituent proteins, which are commonly referred to as nucleoporins (Nups). The core structure appears to be conserved and consists of several Nup subcomplexes, which combine in multiple copies to produce an arrangement of eight asymmetrical units, referred to as spokes, which are positioned symmetrically around the axis of rotation [262]. The Y complex, which is synonymously referred to as the Nup107 complex, constitutes the primary component of the outer rings, comprising the nuclear ring (NR) and the cytoplasmic ring (CR), which are positioned remotely in the nuclear and cytoplasmic areas. The inner ring complex provides the framework for the inner ring (IR), which is synonymous for the spoke ring and is located at the fusion interface of the nuclear membranes. The spoke ring comprises the scaffold proteins Nups 35, 155, 188, and 192, along with the Nsp1 complex. The IR constitutes a centralized tunnel covered with phenylalanine-glycine (FG) repeats that harbor nups engaging with cargo complexes. The Nup159 complex, which is synonymously with the P complex, attaches itself asymmetrically to the Y complex of the CR and facilitates the export of mRNA.

NUP155 is a conserved protein and is necessary for the fusion of the NE membrane and for the construction of the NPC [263]. NUP155 performs a key part in the development of the new NE after cell division and is involved in the attraction of other nuclear pore proteins. It is crucial for its structure, function, and the regulation of the bidirectional macromolecule trafficking of macromolecules across the pores between the cell nucleus and cytoplasm. Moreover, NUP155 performs a pivotal role in mechanotransduction by connecting the cytoskeleton to the nucleus, perceiving and propagating mechanical cues, and regulating the integrity of the nuclear envelope and protein trafficking. Mutations affect heart functioning (atrial fibrillation) and cell viability, underscoring its significance for force transduction and cellular reactions to physical stimuli. Interactions with other nucleoporins (such as Nup93) and possibly lamin proteins enable NUP155 to span the distance between the cytoskeleton and the nucleus, thereby facilitating the transfer of mechanical forces. Force is transmitted via external mechanical stimuli, which are conveyed from the plasma membrane through integrins and actin filaments and the LINC complex into the NE, where NPCs, such as NUP155, serve as important switching points and influence the structure and functionality of the cell nucleus. Moreover, NUP155 fulfills a specific role, as it impacts the activity of YAP. NUP155 interfaces with the mechanosensitive transcription factor YAP and regulates its localization in the cell nucleus and its activation in reaction to mechanical signals originating from the ECM. Consequently, NUP155 functions as an important sensor and transducer at the NE by converting mechanical stresses into biochemical cues that drive cellular processes. Thus, NUP155 is crucial for preserving the integrity of the nucleus and cellular reactions against physical forces.

#### 5.2.2. How Can NPCs Contribute to Nuclear Mechanotransduction?

**Figure 5:**
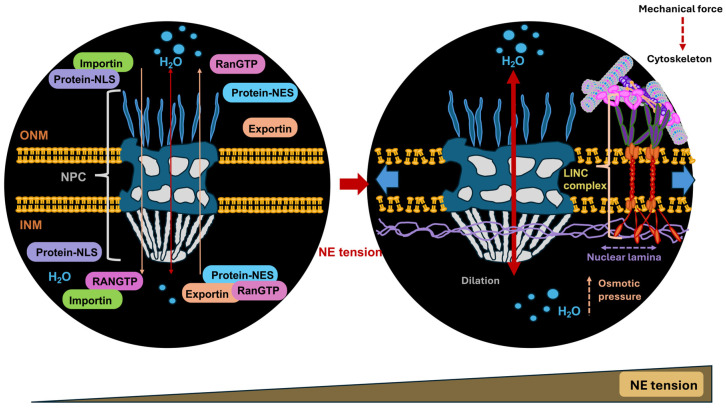
Tension-regulated opening of the nuclear pore complex and tension-controlled transport in and out of the nucleus. Nuclear trafficking is carried out by nuclear shuttling transport receptors (NTRs/importins/exportins), which detect NLSs or nuclear export signals (NESs) within macromolecules and shuttle them via NPCs. Ran-GTPase occurs in a GTP-bound state in the cell nucleus, where it supplies energy and direction for transportation through liberation of NTR-NLS complexes and reinforcement of NTR-NES complexes. The tension (red arrow) on the NE and nuclear lamina is modified through intracellular and extracellular signals that change the osmotic and/or direct mechanical forces applied to the NE and LINC complexes. The tension of the NE expands the NPCs, rendering them increasingly permissive to water and potentially contributing to a reduction in the efficacy of the NPC diffusion restriction, resulting in a heightened efficiency of passive transit for relatively small macromolecules (blue circles).

Thousands of NPCs are located on the surface of mammalian cell nuclei, serving as selective transportation channels that biochemically partition the nucleoplasm from the cytoplasm. Although the molecular composition and structure of archetypal NPCs are fairly well characterized, different cell types and even individual cells feature dissimilar NPCs that consist of distinct nucleoporins. In addition, the integration of NPCs into mechanosensitive networks impacts their state of dilation. Nevertheless, it remains unclear whether (and how) the dilatation (opening, expansion) or the plastic composition of NPCs contributes to their primary function as selective transport channels. Based on the present knowledge of the plasticity of NPCs, a hypothesis is proposed here that the tension of the nuclear membrane and the resulting dilatation of the nuclear pores is a crucial determinant of the plastic composition of NPCs (Figure 5) [264]. This offers a context for interpreting how nucleoporins can determine the fate of cells and sheds light on the tissue specificity of some NPC-related diseases.

How is the influx and outflux of substances through NPCs induced via mechanotransduction? Tensile stress intensified the polymerization and elongation of F-actin, thereby enhancing the expression of the LINC complex. These actions further strengthened the tension on the NE, expanded the nuclear pores, and promoted the transportation of YAP into the nucleus, thereby amplifying the expression of proliferation-related genes. During this process, mitochondria are moved along the reconstituted microtubules toward the edge of the cell nucleus. They produced ATP to drive the nuclear translocation of YAP and promoted F-actin polymerization to a certain extent. Destruction of the LINC complex impaired nuclear translocation of YAP, which restricted proliferation of periodontal ligament stem cells (PDLSCs), hindered reshaping of periodontal tissue, and prevented tooth movement [265]. Similarly to YAP, the LINC complex is linked to the nuclear localization protein β-catenin, which is key in controlling how stem cells grow and stay active [266,267]. Mechanical stress induces a transformation of the cell nucleus and the creation of transmembrane actin nuclear (TAN) lines with linked NPCs. The response of the cell nucleus to mechanical stimulation through employment of uniaxial cyclic stretching has been explored using fluorescence microscopy and quantitative image analysis. It has been revealed that the stretch-induced lengthening and alignment of the cell nucleus orthogonal to the stretch vector relies on formin-guided actin polymerization [268]. The mechanosensitive transcription factors YAP/TAZ and myocardin-related transcription factor (MRTF-A, alternatively designated MKL1 and MAL1) concentrate in the cell nucleus and trigger the activation of target genes upon exposure to uniaxial cyclic stretch. TAN lines have been found to be triggered by stretching and NE proteins like nesprins, SUN2, and laminins form LINC complexes that line up with actin stress fibers [268]. These NE structures are disrupted by pharmacological treatments (cytochalasin D and jasplakinolide) or genetic perturbations (zyxine gene deficiency) that change actin, and these perturbations remain stable as long as the stretch stimulus is sustained [268]. NPCs are concentrated along TAN lines and offer a plausible mechanism for connecting mechanical signals to the functionality of NPCs. How is the nuclear trafficking and signaling controlled by mechanical forces? NPCs, macromolecular complexes that span the nuclear membrane and control the trafficking of RNA and proteins into the nucleus, are equally sensitive toward mechanical force [269,270]. These structures can expand when mechanical forces are applied, taking on a more wide-open shape, which increases the import of transcription regulators like YAP [253]. Nuclear transportation can be additionally controlled by mechanically unfolding proteins to increase their movement into the nucleus, which results in alterations in gene expression [270]. Ultimately, the phosphorylation of nuclear constituents such as lamin A/C or emerin is also modified by mechanical forces exerted on the nucleus, although the molecular mechanisms involved are still uncertain. The phosphorylation of lamin A/C is induced upon low cytoskeletal tension on soft adhesive substrates, causing enhanced movement and turnover of lamin A/C [145,193,271]. In contrast, the phosphorylation of emerin is induced in response to high tension, leading to enhanced interactions between the lamina and the cytoskeleton and to mechanosensitive signal transduction involving YAP [272]. Newly synthesized emerin is incorporated post-translationally into the ER and then diffuses across the ER into the NE [273,274]. Emerin passes into the cell nucleus through passive diffusion, while remaining tethered to the membrane [274,275]. Its positioning is supported through its connection to lamin proteins of type A. Emerin, together with Lap2ß and MAN1, is among the earliest identified members of the LEM domain protein family. The LEM domain of emerin is positioned at the residues 4 to 44 at its N-terminus and interferes with barrier to autointegration factor (BAF) [276,277]. BAF can concurrently connect to emerin and DNA. Apart from the LEM domain and the residues 223 to 246 that form the transmembrane domain, emerin lacks a well-defined secondary structure [278]. Emerin residues 70 to 178 connect to lamin A [279] and facilitate its concentration at the NE [280]. Since both emerin and lamin A are not linked to assembling NE assembly in cells expressing a dominant mutant of BAF, it has been assumed that BAF can be crucial for NE localization of emerin [281]. BAF is therefore an critical NE localization component for both emerin and lamin A following mitosis [281]. Collectively, these results imply that emerin recruitment and maintenance may require sequential engagements with BAF and lamins [281]. Emerin controls the cell nucleus’s architecture and robustness. Its absence impairs the connection between the nucleoskeleton and cytoskeleton, resulting in softer, more malleable cell nuclei [282]. Reduced expression of emerin and the resulting deterioration in nucleoskeletal attachment are connected to the advancement of cancer, especially in breast, ovarian, and prostate cancer [283]. Emerin contributes to the stabilization of RhoA activation in the posterior part of the cell nucleus, which is crucial for cell polarity and migration through narrow areas (restricted migration). It also interfaces with histone-deacetylase 3 (HDAC3) and other proteins to regulate chromatin organization and gene expression in reaction to mechanical cues [284].

### 5.3. Cytosolic Phospholipase A2 (cPLA2)

At first glance, mechanotransduction in stretched membranes is frequently viewed as synonymous with the activation of mechanosensitive ion channels, but there are a multitude of other mechanisms that are facilitated through the unfolding of membranes, membrane tension, bending of the membrane, or reorganization of membrane domains [285]. Reconstituted systems demonstrated that peripheral membrane enzymes are also reactive to the tension of the bilayer [286,287,288]. Wound healing experiments with zebrafish revealed that cPLA2 acts as an important mechanosensor at the INM [289,290]. The induction of a local osmotic stress and cytosolic swelling is concomitant with the trafficking of cPLA2 toward the INM [290]. cPLA2 directly converts physical forces at the cell nucleus into biochemical inflammatory cues. The mechanism of mechanotransduction works as described in the following. The cell nucleus acts as the primary mechanotransduction organ in this process. Mechanical stimuli like osmotic stress, cell confinement, or tissue injury cause the NE to stretch. Increased tension on the INM causes cPLA2 to move from the nucleoplasm toward the NE. This process is physically determined. While calcium (Ca^2+^) is necessary for binding, it is not sufficient on its own; the mechanical tension of the membrane is the decisive trigger for full enzymatic activity. As soon as cPLA2 is active at the stretched membrane, it initiates a cascading process. cPLA2 breaks down phospholipids in the sn-2 position to liberate arachidonic acid. Arachidonic acid is converted directly at the NE into proinflammatory eicosanoids such as leukotrienes via enzymes like 5-lipoxygenase (5-LOX). These signals govern immune responses, such as neutrophil chemotaxis at wound sites, cell proliferation in keratinocytes, and metabolic adaptations in vascular muscle cells. Thereby, the ER typically functions as a buffer, supplying membrane material to maintain low tension at the NE. When this buffer capacity is completely used up or the ER is disturbed, the tension rises sufficiently to activate the cPLA2-controlled mechanotransduction. These insights confirmed cPLA2-dependent nuclear membrane mechanotransduction (NMMT) as a mechanism for wound perception that drives the conversion of osmotic swelling toward inflammation.

**Figure 6:**
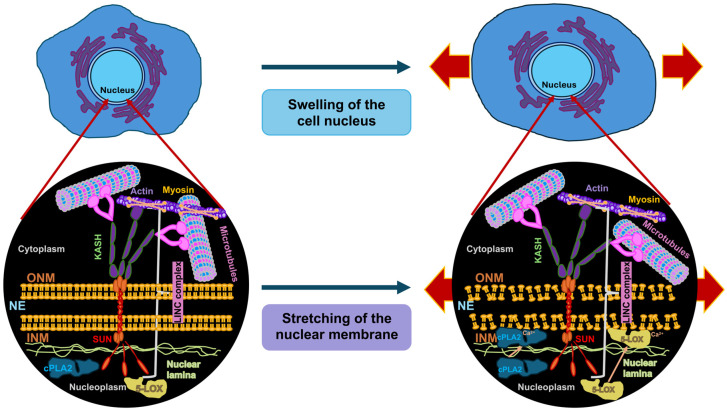
The membranes of the NE are supported by the protein lattice of the nuclear lamina and the cytoskeleton, which are connected to each other via the LINC complex. Stretching forces caused by mechanical disturbances like swelling of the nucleus are passed on to the protein meshwork of the lamina and the cytoskeleton, as well as to the nuclear membrane. Stretching of the nuclear membrane causes an elevation in membrane tension within the plane and results in loosening of the lipid arrangement, which facilitates new hydrophobic protein–lipid connections between the inner nuclear membrane and proteins like cPLA2 and 5-LOX in the presence of Ca^2+^ ions.

How can cPLA2 integrate into the lipid bilayer of the cell nucleus? PLA2s such as cPLA2 catalyze the hydrolysation of the sn-2 acyl chain of phospholipids to produce free fatty acids and lysophospholipids. For this purpose, PLA2 enzymes insert hydrophobic residues in their catalytic domain and/or specific membrane-binding motifs in the lipid bilayer [291], thereby PLA2s are turned into effective membrane-bound stress sensors [292,293]. The absorption of PLA2s is impaired by densely packed headgroups [287], while less densely packed bilayers exhibit hydrophobic lipid packing defects (LPDs) that function as adsorption spots. Positive curvature [294,295,296], conical lipids [297], and mechanical stretch stretching can facilitate the formation of LPDs. Since the nuclear membranes are relatively flat, the membrane tension usually prevails the formation of LPDs. cPLA2, like mechanosensitive channels, senses the tension of the lipid bilayer through hydrophobic membrane interactions in the form of “force from lipids.” Disturbance of the constitutively nuclear localization of cPLA2 in zebrafish impairs confinement perception [298], suggesting an accumulation of one or both of its activating signals, Ca^2+^ or tension/LPDs, in the deformed nucleus. Since Ca^2+^ is swiftly balanced through the nuclear pores [299], the INM tension is probably a key factor. In theory, at least, nuclear cPLA2 is thus able to differentiate between chemical signals such as GPCR or purinergic receptor agonists, which raise Ca^2+^ levels but leave LPDs unchanged, and mechanical stimulation of the cell body, which raises both Ca^2+^ levels and increases LPDs. A physical-chemical AND-gating feature could let cells collect complex proprioceptive signals from their physical surroundings [298]. Specifically, migrating cells utilize the cell nucleus as a “yardstick” to gauge both the dimensions of their immediate microenvironment and sudden alterations in their own volume resulting from external forces, like physical constraints [111,219] (Figure 6). For instance, migrating leukocytes use cytoskeletal forces to extrude their cell nuclei into several nearby connective tissue pores to assess pore sizes. Subsequently, the cell selects the pores with the largest size as the route for cell migration [219]. The suggested ruler-like mechanism is delightfully straightforward: the cell nucleus can undergo deformation so that the membrane reservoirs of the ONM are completely unfolded. Each subsequent deformation causes the ONM and probably the adjacent ER to stretch and tense, which then activates stretch-induced calcium channels and consequently results in downstream responses such as increased actomyosin contractility [111].

A analogous mechanism of nuclear membrane deformation occurs in epithelial monolayers in reaction to the stretching of the substrate [109]. As a result, stretch-activated calcium signaling, which is activated by the stretching of the nuclear membrane through the Piezo-1 ion channel, decreases the chromatin occupancy of H3K9me3 throughout the genome, leading to a softening of the nucleus, which thereby shields it from mechanical harm. Nuclear deformation caused by cell expansion or osmotic swelling of the nucleus has also been found to induce perinuclear calcium liberation, leading to increased calcium levels inside the nucleus that alter expression of genes [300]. The osmotic swelling of the cell nucleus causes cPLA2 to become activated by encouraging its insertion into the hydrophobic membrane, which, combined with elevated calcium levels, increases inflammatory signals and the contractility of the cell [111,243] (Figure 6).

If presence in the nucleoplasm is necessary for mechanosensitivity, how is the import of cPLA2 into the nucleus controlled? Human cPLA2, whose nuclear localization signal (NLS) motifs are less effective, is largely located in the cytoplasm and is able to translocate to the cell nucleus under certain conditions, for example, in tissues that are subject to mechanical stress, such as urothelium, skin, and uterine smooth muscle [301]. Since nucleocytoplasmic trafficking is also influenced by the physical and chemical features of the cargo [270,302], it appears reasonable to explore whether differences in the mechanical strength of NLS-proximal protein regions, possibly in conjunction with post-translational modifications, could be responsible for differences in subcellular cPLA2 localization. The nuclear trafficking of many signaling transducers, for example, YAP, has been found to be mechanically driven [253,303], and nuclear cPLA2 accumulates in dendritic cells under conditions of restriction [304]. Moreover, the nuclear swelling causes an incorporation of cPLA2 and 5-LOX in the INM (Figure 6) [290]. It remains to be clarified whether human cPLA2, which has a larger size compared to YAP, is also subject to mechanical control through facilitated trafficking via nuclear pores. The idea of a second mechanical gatekeeper prior to INM adsorption is appealing. Notably, both cPLA2 and Ca^2+^-independent iPLA2 accumulate in the nucleus following caspase-independent cell death in response to hypoxia [305]. Dying cells display marked nuclear deformations with increased INM tension [306,307]. Nevertheless, it remains uncertain whether and how the mechanical membrane stress associated with cell death/cell injury controls the transportation of cPLA2 into the cell nucleus. Cancer cells move mesenchymal on flat substrates, but in confined spaces they shift to a bleb-based amoeboid mode of locomotion, which is triggered when the nucleus is compressed [308,309]. Alongside other NMMT-independent mechanisms [310], cPLA2-NMMT is therefore a key driver of this transition from mesenchymal to amoeboid cells (MAT) [111,298]. In type of cancer cell migration, the position of the nucleus at the cell’s rear is decisive, as it aids in pushing the cell forward in a piston-mechanism-based migration mode [226,311], as reviewed in [147]. cPLA2 releases arachidonic acid out of the nuclear membrane, thereby activating myosin ATPases [111,298]. The cPLA2 signaling cascade interacts with other mechanisms including RhoA-Rho-associated protein kinase (ROCK) signal transduction and Ca^2+^ influx (typically through Piezo1) to increase cell contractility. Piezo1 promotes cell migration via Ca^2+^-driven activation of phosphodiesterase 1 (PDE1) [310]. In addition, Piezo1/Ca^2+^ stimulates inverted formin-2 (INF2) to initiate extensive reorganization of the actin cytoskeleton. It is noteworthy that INF2 activation promotes detachment, which in turn promotes mesenchymal-amoeboid transition (MAT). Using microfabricated surfaces, cells necessitate INF2 to efficiently migrate in settings with demanding mechanochemical characteristics [310]. Thus, the MAT transition, which is frequently observed in melanoma and other cancers, enhances the plasticity of cells and enables more efficacious movement and metastasis even under challenging circumstances like confinements, as reviewed in [312].

### 5.4. Nucleoskeleton

The nucleoskeleton is a pivotal internal scaffold of the cell nucleus that constitutes a network extending throughout the entire nucleus, including the peripheral and central parts. The nucleoskeleton consists of the following main components: There is the lamina that comprises a peripheral layer of lamin intermediate filaments. Consequently, a dense scaffold is formed that mechanically supports organization of the nucleus and is connected to the INM of the NE. This scaffold offers structural stability, and tethers chromatin. In addition, it supports nuclear organization and essential function, such as replication of DNA, RNA splicing and transcription by providing a mechanical support structure [313]. Specifically, lamin-associated proteins (LAPs), such as proteins like LAP2α, interface with the lamina and are engaged in the process of DNA replication. In addition to intermediate filaments and their associated proteins, actin and actin-associated proteins constitute the nucleoskeleton. They are engaged in remodeling of chromatin, synthesis and transportation of mRNA. Other proteins of the nucleoskeleton include spectrin (mechanical resilience and elasticity), titin (acts as a molecular spring, ensuring passive rigidity), and special nuclear elements such as Skeletor (mitotic spindle organization, especially in Drosophila), the expandable associated structure of the nucleus (EAST; aids in organizing chromatin territories and offers mechanical support to the NE, interacts with nuclear speckles and the nucleolus, to orchestrate RNA processing and assembly of ribosomes), and the nuclear mitotic apparatus (NuMA; directs microtubule organization in assembly of the mitotic spindle, where it recruits dynein motors and interconnects microtubules to strengthen the spindle).

The diverse functions of the cytoskeleton can be summarized in the following four roles: First, mechanical stability provides the basic structure to protect the nucleus from mechanical stress. It connects to the cytoskeleton through the LINC complex and is a key part of keeping the nucleus mechanically stable and its genome intact. Second, the nucleoskeleton acts in genome organization, as it organizes and anchors chromatin, ultimately impacting gene expression. Third, it controls the expression of genes, as it provides a hub for replication, transcription and splicing including alternative splicing. Fourth, the nucleoskeleton ensures connectivity, as the LINC complex bridges the internal nucleoskeleton to the external cytoskeleton. In this context, the nucleoskeleton appears to be of great importance for cell function, as mutations in nucleoskeleton proteins are implicated in a multitude of diseases. In addition, the nucleoskeleton provides structural support for the core components, thereby sustaining the shape of the nucleus and preserving its mechanical integrity, while connecting to the cytoskeleton. In summary, it regulates the genome, as it organizes chromatin, drives DNA replication, transcriptional processes and processing of RNA. Moreover, the nucleoskeleton connects nuclear events with cytoplasmic signaling mechanisms. Essentially, the nucleoskeleton is not merely peripheral, but rather a dynamic internal scaffold that is indispensable for the organization and functionality of the cell nucleus, connecting the NE with the chromatin and other nuclear elements. Nevertheless, although the nucleoskeleton spans the entire interior of the cell nucleus in the form of an internal nucleoskeleton, it is also concentrated at the NE in the shape of a peripheral lamina. Thus, it is still seen here as part of the peripheral elements of the cell nucleus that form the interface between the cell nucleus and the cytoplasm.

### 5.5. Nuclear Lamina (Part of the Nucleoskeleton)

The nuclear lamina is a protein network that is located at the INM, where is provides support for the nuclear membrane and aids in organizing the chromatin within the nucleus. The nuclear lamina comprises mainly Lamin A/C and Lamin B, which are structural proteins. Lamin A and lamin C are alternative splice variants of the exact same gene. Together with other lamin-associated proteins, they create a fibrillar network. It provides stability to the cell nucleus and connects chromatin and nuclear pores. This framework performs a critical function in chromatin organization, gene expression, and mechanical stabilization of the nucleus. It can therefore be assumed that changes in the mechanical properties of the cell nucleus are based on mechanical stabilization by lamins. In summary, exploring the interface between the nucleus and the cytoskeleton is crucial for understanding the mechanical characteristics of the cell nucleus. Moreover, it is highly relevant for the nuclear mechanosensoring and mechanotransduction function.

## 6. Central Nuclear Bodies Act as Mechanosensory Elements That Regulate Mechanosensing and Mechanotransduction

**Figure 7:**
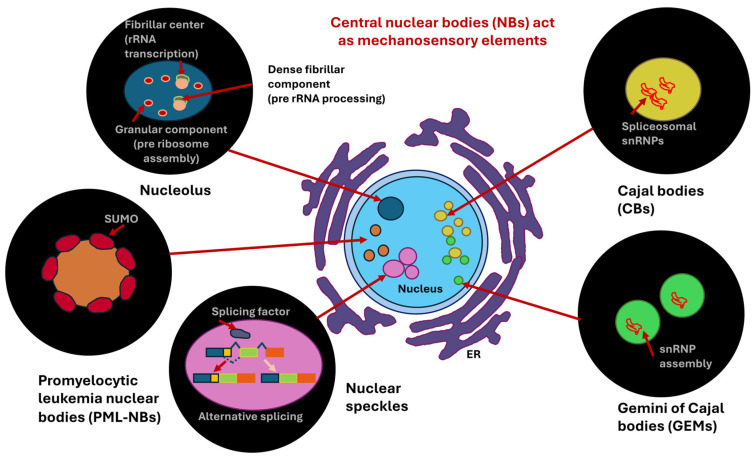
Central nuclear bodies serve as mechanosensory elements, such as nucleolus, Promyelocytic leukemia nuclear bodies (PML-NBs), Nuclear speckles, Gemini of Cajal bodies (GEMs) and Cajal bodies (CBs) that control mechanosensing and mechanotransduction. The alternative splicing takes place in nuclear speckles. At the fibrillar center of nucleolus, the rRNA transcription occurs, the pre-rRNA processing takes place in the dense fibrillar component and the pre ribosomes assembly is carried out in the granular component. The GEMs assembly snRNPs and the CBs contain spliceosomal snRNPs.

Central nuclear bodies function as mechanosensory elements comprising nucleolus, PML-NBs, CBs and GEMs, and nuclear speckles (Figure 7).

### 6.1. Nucleolus

The nucleolus is a dense structure within the cell nucleus that is not enclosed by a membrane. It functions in ribosome synthesis, as it produces ribosomal RNA (rRNA) and forms together with RNA-binding proteins (RBPs), which are a broad group of proteins that specifically bind to RNA molecules, ribonucleoprotein particles (RNPs) via interactions with their RNA-binding domains (RBDs) proteins [314]. The most abundant RNPs are ribosomes. RNPs build also spliceosomes [315] and small nucleolar RNPs (snoRNPs) that chemically modulate and process rRNAs [316]. The nucleoli can also be physically displaced by mechanical input, such as compression and stretching.

### 6.2. Promyelocytic Leukemia Nuclear Bodies (PML-NBs)

PML-NBs are subnuclear structures with a size of 0.1 µm to 1.0 µm and are made up of a PML protein casing. Similar as the other subnuclear structures, they seem to play a role in providing mechanical properties of the cell nucleus. Nevertheless, it needs to be experimentally confirmed that they contribute to nuclear mechanical cues and consequently cancer cell migration and invasion through confinements. PML-NBs are dynamic nuclear entities that function as nodes for stress reactions, protein modification, such as SUMOylation, and gene regulation, affecting cellular processes such as senescence, apoptosis, and defense against viruses [317]. PML-NBs are spheres with a diameter of approximately 0.1–1 μm and are located in most mammalian cell nuclei [318]. How are NB-NBs interacting with chromatin to impact gene expression, control transcription factors, and take part in protein quality oversight? Although PML-NBs themselves cannot be considered as conventional mechanoreceptors, their dynamic characteristics, their function as a protein framework, and their participation in stress signaling enable them to engage with mechanotransduction routes. This may occur through the incorporation of important regulatory factors that react to mechanical signals and impact chromatin, transcription, and whole-cell adjustment to forces. PML proteins create spherical structures in which multiple partner proteins build up, generating functional activity centers. These stress reaction centers participate in the reaction to oxidative stress and other stress reaction routes and control the activity of proteins with SUMOylation. It has been hypothesized that high concentrations of small ubiquitin-like modifier (SUMO)-bound proteins aggregate into liquid-like droplets and that this phase transition can take place inside nuclear bodies like PML-NBs [318]. Moreover, regulation of gene expression is ensured through PML-NBs, which interface with chromatin and affect genes linked to cell cycle, senescence, and cancer suppression. PML-NBs act in mechanotransduction. For instance, mechanical forces can influence nuclear structural elements, and PML-NBs, acting as dynamic nuclear domains, are probably impacted through these forces, similar to other nuclear bodies such as nucleoli. PML-NBs play a central part in cellular stress reactions such as oxidative stress, and mechanical stress is an important cellular stressor. Therefore, PML-NBs are able to integrate mechanical cues into other stress response mechanisms. PML-NBs frequently change expression of genes, as mechanotransduction turns mechanical cues into biochemical reactions. PML-NBs control transcription and are therefore likely places where mechanically triggered cues, such as modified protein SUMOylation or the state of chromatin, come together to manage gene networks. Moreover, important PML-NBs constituents like Death-Domain Associated Protein (DAXX) and α-thalassemia/intellectual disability syndrome X-linked (ATRX) are engaged in chromatin reorganization, and their activity inside the PML-NBs can be impacted through mechanical inputs, thereby having an effect on how the cell adjusts to forces. The ATRX protein is attracted to telomeres through interaction with G-quadruple (G4) DNA structures, whereas the DAXX protein functions as a chaperone that specifically associates with histone variant H3.3. DAXX functions as an H3.3 chaperone and targets PML-NBs via SUMO-interacting motifs (SIMs) that attach to SUMOylated PML-NBs. DAXX is critical for the integration of the non-canonical histone variant H3.3 into telomeres and pericentric heterochromatin. H3.3 acts as a target for H3K9me3, which is key for attracting HP1α and keeping chromatin in a silent state. The lack of H3.3 or its chaperones causes telomere malfunction, elevated DNA injuries, and alternative lengthening of telomeres (ALT) in cancer cells like glioblastoma cells [319]. ATRX acts as a chromatin-remodeling ATPase that combines with DAXX to build a complex. ATRX connects the DAXX/H3.3 complex with chromatin, thereby promoting H3.3 deposition [320]. In the general case, DAXX controls the deposition of H3.3 in heterochromatin, like pericentromeres, retrotransposons, and subtelomeric regions, while histone cell cycle regulator (HIRA) participates in the deposition of H3.3 within euchromatin, like actively transcribed gene loci and regulatory sequence elements [321,322]. The DAXX/ATRX complex is typically associated with the maintenance of genome stability. Similar as the nucleolus, the PML-NBs can be physically displaced via mechanical inputs. Finally, PML-NBs function as critical cellular signal integration hubs. Their capacity to adapt quickly under stress, attract signaling molecules, and alter chromatin suggests that they are ideally placed to sense and transmit mechanical cues, thereby integrating the mechanical microenvironment of the cell with its key processes, such as cell survival, proliferation, and fate determination.

### 6.3. Cajal Bodies (CBs) and Gemini of Cajal Bodies (GEMs)

CBs are small, dynamic, membrane-less spherical structures with 0.5 to 1.0 µm in diameter that are associated with the biosynthesis of ribosomes and processing of RNAs and the maturation of RNPs [323]. Multiple proteins participate in the assembly of CBs, among them Nopp140 (NOLC1), WRAP53, and Coilin. Coilin is altered by SUMO at several distinct lysine residues [324]. The abundance of CBs tends to differ based on cell type, stage of development, or level of transformation [325]. Transformed cell lines such as HeLa, for instance, exhibit a higher proportion of cells with CBs compared to non-transformed cells like WI-38 [326]. The immortalization of cell lines can also influence the quantity and function of CBs. In addition, in a neuroblastoma cell line, SH-SY5Y, the differentiation of SH-SY5Y is linked to an increase in CBs [327]. To investigate this point more closely, additional experiments on non-transformed cancer cells are therefore necessary. It also needs to be clarified whether the presence of CBs is related to the migration and invasion of cancer cells. CBs serve as mechanical sensors. Physical forces or mechanical stresses are known to cause a straightforward, fast breakdown of their key building blocks, like Coilin and the Survival Motor Neuron (SMN) protein. Coilin, which serves as the marker protein for CBs, is regarded as a comprehensive sensor for environmental stimuli, such as mechanical forces. Fluorescence resonance energy transfer (FRET) studies have demonstrated that external forces acting on cells can cause fast separation of SMN from coilin inside the CB, suggesting a direct mechanical reaction [328]. Disturbance of these mechanical cues can result in impaired snRNP and telomerase production, indicating that force directly regulates CBs’ “factory” functionality. Based on these observations, it can be hypothesized that these mechanically altered CBs can change the entire nuclear mechanics and also the cell mechanics themselves, thereby altering the migration and invasive capacity of the cancer cells. Progress in this area is still in its infancy and requires more attention. It is noteworthy that the architecture and functions of these CBs, together with the nuclear spots and nucleolus, are involved in controlling the spatial organization of the genome [329]. GEMs are spherical nuclear organelles measuring 0.1–2.0 µm in size, made up of the SMN complex, which serve as essential locations for storing or assembling spliceosomal snRNPs. GEMs are frequently detected nearby CBs. They can be clearly distinguished from CBs, as are devoid of coilin. In particular, GEMs are implicated in the assembly and alteration of spliceosomal snRNPs (small nuclear ribonucleoproteins), which are important for pre-mRNA splicing. GEMs are indispensable for snRNP assembly, and their loss has been associated with spinal muscular atrophy (SMA).

### 6.4. Nuclear Speckles

Nuclear speckles refer to dynamic, fluid-like domains in the cell nucleus and are irregularly shaped, droplet-like molecular condensates that harbor a variety of splicing factors. What are the major effects of the mechanical properties of nuclear speckles? All molecular condensates consist of SON and SRRM2 proteins, and non-coding MALAT1 RNA encircles these condensates [330]. The nuclear speckles act as dynamic condensates. Specifically, nuclear speckles arise by liquid–liquid phase separation (LLPS) and act similar to liquid droplets, where constituents (splicing factors, lncRNAs such as MALAT1) are continuously shuttled with the nucleoplasm, enabling fast rearrangement. In addition, the non-coding RNA MALAT1 trapped in the cell nucleus controls alternative splicing by changing how SR splicing factors are phosphorylated [330]. Nuclear speckles interact with chromatin. Nuclear speckles travel to active genes and bind to them, thereby boosting transcription and splicing. Their positioning is affected both through gene activity and nuclear scaffold architecture. Nuclear speckles are subject to structural alterations. For example, under stress or transcription inhibitory conditions, speckles can merge and become condensed, thereby altering their form and size. This process is regulated through proteins such as SON and long-non-coding RNAs (lncRNAs) and influences the overall organizational structure of the cell nucleus. Nuclear speckles can act in mechanotransduction processes. Notably, forces originating from the cytoskeleton can impact the cell nucleus, and nuclear speckles, as fluid condensates, reshape the chromatin matrix and are themselves impacted through these forces, resulting in mechanical cues being coupled to gene expression. Beyond this, there exist molecular interplays of proteins inside speckles, such as SRSFs and TDP-43, which generate specific structures and microphases propelled by intrinsically driven interactions and RNA connections (such as MALAT1), which determine the structure and functionality of nuclear speckles. They serve as repositories and sites of assembly for the RNA processing apparatus and modulate gene expression through movement and merging in accordance with cellular demands and stresses, engaging chromatin and the ambient nucleoplasm, and experiencing structural alterations that couple their mechanical characteristics directly to the control of transcription and the metabolism of RNA [331,332]. Especially in the ordinary interphase of the cell cycle of multicellular organisms, these condensates are widely dispersed throughout the entire cell nucleus. Conversely, when cellular transcription is shut down, the condensates merge and turn into super-compact spherical droplets. Their mechanics encompass LLPS and interplay with chromatin, enabling them to rearrange themselves, amplify gene expression in proximity to active genes, and react to mechanical stimuli. What are their effects on cellular functions? They govern the splicing process by storing and modifying splicing factors, thereby influencing the splicing effectiveness of certain transcripts, particularly those with introns that are challenging to excise. In addition, they respond to stress, as speckle dynamics and localized distribution vary throughout cellular stress such as heat shock, indicating a connection to stress-induced gene expression patterns. Abnormalities in the structure and functioning of nuclear speckles appear to be closely linked to certain diseases, like cancer. For instance, abnormal speckles indicate a change in localization inside the cell nucleus, increased concentrations of the TREX RNA export complex, and are associated with worse patient outcomes in clear cell renal cell carcinoma (ccRCC), which is a type of cancer characterized by hyperactivation of the transcription factor HIF-2α [333]. Moreover, HIF-2α facilitates the physical interaction of specific target genes with speckles, based on HIF-2α protein speckle targeting motifs [333]. Homologous speckle-targeting motifs have been observed in numerous transcription factors, implying that DNA speckle targeting could be a universal mechanism of transcriptional regulation [333]. Through the integration of functional, genomic, and imaging investigations, it has been determined that the gene regulatory programs of HIF-2α are influenced by the speckle state and by the abolition of HIF-2α-controlled speckle targeting. These findings indicate that an essential biological role of nuclear speckles in ccRCC involves regulating the expression of selected HIF-2α-regulated target genes, which in turn affect patient disease progression. Apart from ccRCC, tumor speckles largely relate to changes in functional routes and the expression of speckle-associated gene environments, highlighting a general connection between nuclear speckles and gene expression deregulation in human cancers. In summary, nuclear speckles fulfill functions in both normal cellular conditions and disease. In summary, nuclear speckles are highly dynamic. The nuclear speckles’ physical behavior and mechanical interplay with various nuclear constituent are crucial to governing gene expression and reacting to the extracellular environment.

## 7. Nucleoplasm and Chromatin Act as Crucial Mechanosensory Elements

### 7.1. Nucleoplasm Acts as a Mechanosensor

The nucleoplasm is a jelly-like substance that contributes as a filling material to the mechanical characteristics, such as viscoelasticity, of the nucleus. This substance serves as a dynamic, gel-like environment that is vital for the regulation of genes and the structure of the nucleus. The nucleoplasm is mainly composed of water and acts as a carrier for molecules, enzymes, such as DNA and RNA polymerases, metabolites and ions like calcium, potassium, and sodium ions to diffuse and become moved around. The nucleoplasm provides the setting for critical nuclear processes, including the replication of DNA, the repair of DNA damage, the transcription of RNA, and the control of gene expression. The nucleoplasm and mechanotransduction are closely linked, because the nucleoplasm, which is the liquid gel mass in the cell nucleus, and the chromatin it holds, function as a viscoelastically structured entity that perceives, conveys, and reacts to physical forces. The nucleoplasm permits the nucleus to deform and preserve its shape, which can be induced by external mechanical signals, simultaneously sustaining its biochemical operations [234]. The nucleoplasm acts thereby as a mechanosensor: Along with the NE and nuclear lamina, the nucleoplasm acts as the secondary sensory interface of the cell, whereby the first sensory interface is formed between with the external environment and the plasma membrane, enabling it to receive and process physical cues. Nucleoplasm is frequently characterized as a softer, viscoelastic framework in contrast to its chromatin content. The nucleoplasm is equipped with a “mechanical memory,” which implies that the consequences of mechanical forces, like elevated transcription, can endure for several tens of minutes subsequent to the withdrawal of the force [47]. The cellular equivalent of the nucleoplasm is the cytoplasm, both of which contain scaffolding elements, such as actin fibers. Notably, the nucleoplasm comprises a reservoir of G- and F-actin, which undergoes polymerization when exposed to mechanical signals to govern the movement of the nucleus, the organization of chromatin, and the repair of DNA damage. There is a network of protein fibers referred to as the nuclear matrix (or synonymously fibrillar matrix) that assists in arranging the genetic content and preserving structural stiffness. Consequently, the nucleoplasm offers an ideal environment for the cell nucleus to operate as a kind of operations center, where it coordinates the trafficking of substances and guarantees the integrity of the genetic material. A key reason for this is that the nucleoplasm behaves on smaller length scales more like a liquid rather than a solid, is highly permeable to the passage of small objects, and exhibits a nanoscale viscosity comparable to that of water. Transport is slightly decelerated over longer length scales, and large objects, such as large nuclear bodies, travel very sluggishly because they are encased in the chromatin scaffold that hinders their movement [234]. Chromatin is the most central part of the nucleus. It is described in the following section, as it plays a special key role in mechanosensing and mechanotransduction of the cell nucleus.

### 7.2. Chromatin Impacts Nuclear Mechanical Characteristics

The genetic material chromatin is composed of DNA that is tightly wrapped around histone proteins, when in a condensed state. Beyond a passive storage entity, it is a highly dynamic complex of DNA, RNA, and proteins (especially histones) that coordinates important processes in the cell nucleus. Several dynamic processes, like transcription, RNA splicing, and DNA injury repair, are locally partitioned around chromatin to perform specific activities on the genome. In and around chromatin, there is a large number of membrane-less structures, referred to as biomolecular condensates, comprising nucleoli, PML-NBs, CBs, GEMs and nuclear speckles. All this occurs through the physical mechanism of LLPS and associated phase transitions [178,334,335,336,337]. Due to their deformable, fluid-like characteristics, biomolecular condensates are sensitive to mechanical stress: they are capable of dynamically reorganizing themselves through gravity-induced coalescence and can split or even disintegrate under the action of pulling forces [338,339,340]. The process of cell differentiation is governed through epigenetic modifiers and sensors, such as the methyl CpG binding protein 2 (MeCP2), which is a chromosomal protein that attaches to methylated DNA. The level and mutations of MeCP2 lead to a neurological disorder, which is referred to as the Rett syndrome. While differentiation is taking place, the majority of the genome is tightly packed into heterochromatin, whose primary purpose has simply been considered to be gene silencing. Nevertheless, the modifications in gene expression observed in mutations resulting in Rett syndrome could not be utilized as predictive indicators of disease seriousness. MeCP2 has been demonstrated to enhance nuclear stiffness in a concentration-dependent fashion and in dependence on its capacity to cluster heterochromatin throughout differentiation [341]. The MeCP2-dependent enhancement in stiffness cannot be accounted for by alterations in the expression of mechanobiological genes. Instead, it has been determined that it is perturbed by Rett syndrome mutations and corresponds to the clinical severity of the disorder. These results underscore the influence of chromatin architecture on the mechanical characteristics of the cell as an alternative or supplementary mechanism to alterations in cytoskeletal constituents.

### 7.3. Direct and Indirect Impact of Chromatin on Mechanosensing and Mechanotransduction

**Figure 8:**
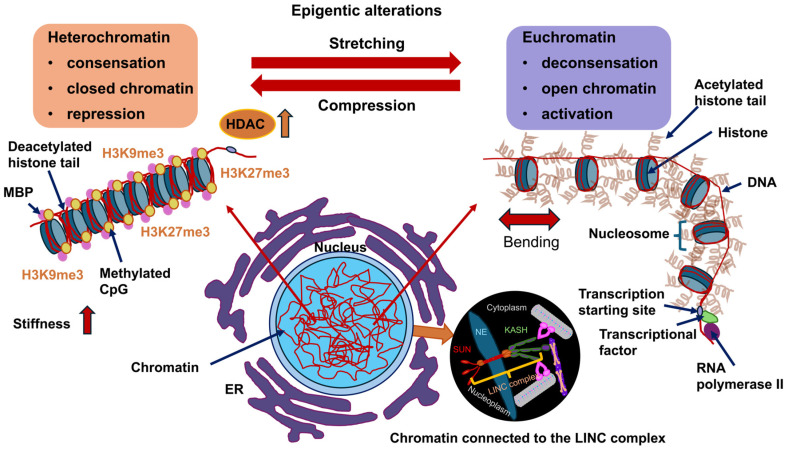
Mechanical cues like tension or compression are conveyed toward the cell nucleus via the LINC complex. Chromatin exists as densely packed heterochromatin and loosely packed euchromatin. Heterochromatin is characterized by DNA methylation and deacetylated histones. It is condensed and transcription factors cannot bind to it, which is why it is referred to as a closed chromatin conformation and represents a repressive control of transcription. In contrast, euchromatin is transcriptionally active, as transcription factors and RNA polymerase II can bind to the transcription start site. The DNA of euchromatin is unmethylated and the histone tails are acetylated, leading to an open chromatin conformation. On the one hand, the chromatin can lead to a switch from heterochromatin to euchromatin through stretching of chromatin, which is connected to the nuclear lamina. Particularly in high-amplitude scenarios, this stretching can lead to a quick decline in heterochromatin markers like H3K9me3. It is assumed that this conversion safeguards the genome from harm by loosening its densely packed structure. Moreover, even nucleosomes can be bent. On the other hand, the chromatin can switch from euchromatin to heterochromatin through mechanical compression. Thereby, the actomyosin contractility is decreased, which triggers the transport of HDAC3 into the cell nucleus. HDAC3 eliminates acetyl groups, which holds chromatin in an open state, and promotes the attachment of methyl groups, thereby enhancing heterochromatin modifications like H3K9me3 and H3K27me3. Chromatin condensation enhances nuclear stiffness and results in reduced overall gene expression (e.g., gene silencing).

Chromatin has a pronounced influence on mechanotransduction as it functions as a dynamic sensor and mechanical force transducer. Chromatin controls the function of the cell through its ability to change its condensation (heterochromatin versus euchromatin) and stiffness in reaction to external stresses, which in turn controls gene expression, nuclear morphology, and long-term fate of the cell, creating a bidirectional feedback circuit with the cytoskeleton and NE (Figure 8).

How reacts chromatin to mechanical forces (outside-in signaling)? Chromatin is involved in mechanosensing and mechanotransduction. Mechanical signals, such as tension or compression exerted by the ECM and cytoskeleton, are conveyed to the cell nucleus through the LINC complex (SUN/KASH proteins). These forces cause alterations in chromatin structure, which are frequently accompanied by histone modifications and chromatin compaction and influence the accessibility of genes. As a consequence, the nuclear mechanical properties are impacted. For instance, elevated compaction (increased heterochromatin) stiffens the cell nucleus and renders it more robust against deformation, thereby safeguarding it from damage. Modified chromatin states (such as euchromatin) result in the transcriptional activation of specific genes such as those in the Rho/Rock signaling cascade and affect the dynamics of the cytoskeleton by promoting actin polymerization and the adhesion of cells. How influences chromatin mechanical cues (inside-out signaling)? The physical state of chromatin has a direct influence on the mechanical characteristics of the cell nucleus and affects cell firmness and stress responsiveness. Condensed chromatin can result in reduced transcription of adhesion genes, which encourages weaker cell adhesion and therefore cell motility, whereas decondensed chromatin encourages increased adhesion. There exists mechanical memory. For example, alterations in chromatin structure can serve as a “memory” for prior mechanical experiences, enabling robust, long-lasting cellular reactions and plasticity in the cell phenotype. What are the key interacting and connecting elements? There is the LINC Complex that connects the cytoskeleton and the NE, and subsequently transmits forces. The NE proteins, lamins act together with chromatin to provide structural integrity.

It is widely established that the genome is physically divided into euchromatin, which is situated in the central region of the cell nucleus, and heterochromatin, which is positioned at the periphery of the cell nucleus [342]. Euchromatin comprises the transcriptionally active fraction of the genome, which accumulates modifications of the histone H3 tail, such as acetylation like histone 3 lysine 27 acetylation (H3K27ac) or trimethylation like H3K4me3 and H3K36me3. Heterochromatin is subdivided into constitutive heterochromatin, which has a more compact structure and is lower in gene density and is marked through dimethylation like H3K9me2 and trimethylation like H3K9me3, and facultative heterochromatin, which can quickly transition to an active transcriptional condition, such as H3K27me3. Modern high-throughput molecular approaches have revealed that eukaryotic genomes are arranged hierarchically and comprise different structural entities marked through specific protein interactions, ranging from nucleosomes to chromatin compartments [343]. These compartments are then broken down into topologically associating domains (TADs) and tiny self-associating regions, showing the complex higher-order architecture of the genome and its incredible flexibility [344,345]. Genomic architecture exhibits high dynamics during various biological phenomena such as differentiation, motility, DNA damage repair, gene splicing, and cell division [346,347,348,349,350,351,352]. How distinct stages of chromatin organization work together to impact gene expression remains an ongoing area of research. In addition, it is uncertain how mechanical cues sensed from the cell nucleus impact epigenomic organization and the molecular processes engaged. Nonetheless, epigenetic modifications have been investigated, with a focus on 3D genome architecture and nuclear structures as mechanisms of reaction to mechanical cues that support cellular memory and adaptive capacity [353]. The remaining question in this area is how chromatin stiffness can impact nuclear mechanics and the capacity of cells to exert forces on their surrounding environment. It has been demonstrated that direct stretching of chromatin enhances the diffusion capacity of proteins and RNA polymerase II, thereby enhancing the transcription of genes. The tension of the nuclear membrane, which is governed through the nuclear lamina, can manage the liberation of transcription factors and signaling molecules. Ultimately, chromatin is not merely a passive package of DNA, but a dynamic mechanical component that converts physical cues into biological results, thereby regulating the form, functionality, and fate of the cell. Consistent with these findings, mechanical confinement, for example, at the interface between the boundary of the primary tumor and the microenvironment, has been shown to regulate phenotypic plasticity through chromatin remodeling in melanoma cells [354]. Spatial and single-cell transcriptomics revealed that these interface cells acquired a gene program of neuronal invasion, encompassing the adoption of an acetylated tubulin scaffold that shields the cell nucleus from damage throughout migration. The DNA-bending protein high mobility group B2 (HMGB2) has been found to be a restriction-induced regulator of neuronal condition. Additionally, HMGB2 levels are upregulated in constricted cells, and quantitative modeling has demonstrated that the constriction extends the duration of contact of HMGB2 with chromatin, resulting in alterations in chromatin configuration that support the development of the neuronal phenotype. The knockdown of *HMGB2* revealed that it controls the balance between proliferative and invasive behaviors, with constricted HMGB2-high cancer cells growing less but becoming more resilient to drug treatments. Finally, these results suggest that the mechanical microenvironment drives phenotypic transition in melanoma.

### 7.4. Chromatin Plays a Role as a Complex, Semi-Fluid Framework

Chromatin acts as a complex, semi-fluid scaffold that determines the shape and stability of the cell nucleus and influences all aspects of cell function, from fundamental cell mechanics to disease mechanisms such as cancer metastasis. Chromatin works like a fluid-like gel: the chromatin network filled with nucleoplasm functions as a porous matrix that allows small movements and the diffusion of molecules and particles. There occur nuclear deformations at the pore level. When the cell nucleus is subjected to slight stretching (in the micrometer range), the fluid-filled chromatin pores distort and function as a spring. This behavior is affected by the state of compaction and alteration of the chromatin itself. Cell migration across confined regions leads to mechanical deformations of the chromatin scaffold, resulting in embedded nuclear condensates, including nucleoli and nuclear speckles, to distort and merge with each other. Chromatin deformations behave differently in the front compared to the rear of the cell nucleus, with the rear half tending to allow the formation of new condensates [355]. This is demonstrably due to an enhanced chromatin heterogeneity, which leads to a shifting of the binodal phase boundary. Collectively, these results demonstrate how chromatin deformation affects condensate formation and characteristics, potentially facilitating the mechanical perception of cells [355].

Chromatin can also undergo movement. Specifically, both exogenous (ECM matrix environment) and endogenous (internal cytoskeleton) forces can lead to decondensation (opening) or condensation (compaction) of chromatin, depending on the strength, duration, and type of force, thereby shifting gene loci and modifying the expression of genes. There are at least for types of forces that lead to chromatin compaction (condensation). First, there are compressive forces. For example, external compression of cells, resulting from crowded environments or high-density growth, results in elevated activity of histone deacetylase (HDAC), which limits the acetylation of histones, leading to considerable condensation of chromatin and diminished activity of transcription. Second, condensin activity impacts chromatin’s mechanical properties and structure. For example, condensin II complexes apply mechanical force to condense chromatin and thereby compact, primarily during interphase in advance of mitosis or to preserve the structure of heterochromatin. Third, mitotic forces control the movement of chromosomes. Throughout mitosis, the two-way pulling forces of the mitotic spindle, together with the effect of condensins, condense chromatin into clearly defined, stiff structures to guarantee correct segregation of chromosomes. Fourth, physical restraints experienced throughout cell migration, such as during the metastatic spread of cancer cells, can cause the chromatin to become more compact, which contributes to the modification of the shape of the nucleus so that it can squeeze through narrow passage ways. There are three main factors that lead to chromatin decondensation and consequently decompaction (opening). First, there are tensile/shear forces exerted on cells. These exogenous tensile stresses or fluid shear forces acting on the cell surface can cause decondensation of chromatin and “softening” of the cell nucleus, a mechanism hypothesized to aid the cell in mitigating mechanical stress. Secondly, there is a temporary reaction of the cells. In the short term, mechanical forces frequently induce an open (decondensed) conformation of chromatin, enabling the cell to adjust to the stress, whereas prolonged application of high forces can induce other, often silencing chromatin conformations. Third, mechanomemory can take place, when exerted through integrins can induce sustained decondensation and enhanced protein dynamics in the cell nucleus. Consequently, these force-driven changes are not only structural in nature, but also critical for the control of transcription, repair of DNA damage, and stability of the cell nucleus. For instance, decondensation frequently enhances the susceptibility of gene expression, whereas intense, persistent forces can result in damage to DNA. Thus, a alteration in chromatin state can affect the entire stiffness and viscoelastic characteristics of the cell nucleus [162,200,218,356,357]. The idea that chromatin deformations can affect the behavior of associated nuclear condensates is supported by a variety of observations about how condensates are tightly linked to the molecular crowding and mechanical features of their local surroundings. In the equilibrium state, phase separation arises when the molecular concentration exceeds the supersaturation point, C_sat_. Previously published research has demonstrated that osmotic stress, alterations in cell volume, or fluctuations in protein concentrations can trigger or prevent phase separation processes in cells, thereby propelling cells beyond the phase boundary [358,359,360,361,362,363,364]. The size and shape of condensates are also influenced through their mechanical environment. Within the cytoplasm, condensates are influenced by the neighboring cytoskeleton [365], whereas nuclear condensates preferentially form and expand in chromatin regions that are mechanically softer and less dense [159,366]. Monitoring the fluctuating movement of condensates inside chromatin yields a subdiffusive exponent, α ≈ 0.5, that accounts for the anomalous coarsening characteristics and is in line with the Rouse-like dynamics resulting from the chromatin entanglement [366]. Nuclear deformations can cause temporary or persistent condensation or decondensation of chromatin and result in mechanical activation of genes, thereby modifying the level of proteins. Alterations in organization of chromatin consequently alter the mechanical characteristics of the cell nucleus, potentially resulting in auxetic behavior [367]. Auxetic behavior describes an unusual mechanical phenomenon in which a material expands perpendicular to the direction of stretching and becomes thinner when compressed. Subsequently, softening of the cell nucleus has been associated with elevated migration and invasion of cancer cells [368]. Moreover, during the metastatic spread of cancer cells, their nuclei soften during transendothelial migration [369].

### 7.5. Coupling Between Nuclear Mechanics and Condensate Behavior

The close connection between core mechanics and condensate dynamics is an evolving topic in mechanobiology [370]. Liquid–liquid phase separation and accompanying phase transitions have been identified as universal mechanisms in living cells for the development of membrane-less areas or biomolecular assemblies, known as condensates. The boundary interface between two immiscible phases exhibits interfacial tension, which produces capillary forces that can affect the surrounding microenvironment. Therefore, nuclear condensates respond not merely to their mechanical environment, but can also deform and hence reshape chromatin via capillary forces [371,372]. In addition, force transmission can occur over long distances of over 30 µm from condensates to their vicinity, as demonstrated by an experiment using mouse oocytes [373]. This suggests that condensates may react to forces that arise as a result of the highly mechanically active cell migration process, whereby the generation of cytoskeletal forces and compression through narrowings can lead to considerable intrinsic stress and strain [374,375,376]. Specifically, protrusions of cancer cells generate forces ranging between picoNewtons and nanoNewtons as they invade. It is noteworthy that the expansion of the protrusions is accompanied by a gradual elevation in force, in increments of 0.2 to 0.5 nN, which are applied at intervals of 30 s to 6 min. When the protrusion is extended in the micrometer area, the forces exerted by the cell range between 1.1 and 2.2 nN [376]. This cellular mechanism therefore unveils a hitherto uncharacterized dynamics of force production through the formation of invasive protuberances of cancer cells [376]. Beyond this, the question arises as to how the cell nucleus contributes to increasing force generation to enable cell movement. Nevertheless, the exact values of the forces acting on the cell nucleus and the protrusion forces are not available; they should be measured in the future. The cell nucleus is by no means passive, but actively participates in the generation of force for cell migration through its physical connection to the cytoskeleton through the LINC complex, functioning as a mechanosensor and modulating gene expression that influences motility. The cell nucleus is drawn through the retrograde actin flow aided by TAN lines, which link the nucleus to the cytoskeletal framework, adapts to the cell contours, and influences cytoskeletal dynamics through its stiffness/position, thereby facilitating forces for migratory movement in restricted settings or the reception of mechanical signals. There is actin filament-driven pulling on the TAN lines, when the actin filaments move backward, which is referred to as retrograde flow, in the course of cell migration. This flow carries the cell nucleus along with it, primarily toward the posterior part of the cell, which contributes to the generation of polarity. The mechanical characteristics of the cell nucleus, including stiffness, viscoelasticity, deformability, and overall shape, which are controlled by the nuclear lamina, help withstand pressure and enable the cells to move in restricted locations, thereby supporting the equilibrium of forces. Consequently, the cell nucleus functions as a mechanical anchoring device and as a sensor that converts forces from the cytoskeleton into cellular reactions and, through its own structural characteristics, enables the intricate movements necessary for cell migration [377].

Quantitative assessments of dynamic nuclear deformation during cell migration across narrow gaps highlight different phases of nuclear displacement due to restriction, bending of the nuclear lamina, and substantial intranuclear stress. In addition, it has been determined that cells with lamin A/C depletion display enhanced and greater plasticity in nuclear deformation than wild-type cells, while exhibiting minimal alterations in nuclear volume. This result suggests that reduced lamin A/C levels promote migration across narrow spaces by enhancing nuclear deformability and not by increasing nuclear compressibility [374]. How can physical deformation caused by cell migration impact the structure and phase behavior of nuclear condensates? Mechanical deformation of the cell nucleus caused by restricted migration of human breast cancer cells can affect chromatin-embedded condensates. A microfluidic migration assay has been employed to demonstrate that endogenous and synthetic condensates undergo substantial deformation as a result of stresses arising in the ambient chromatin scaffold. These stresses appear in different ways in the front and rear halves of the narrowed nucleus, causing different chromatin arrangements and a preferred phase separation in the rear half. These results may indicate how new compartmentalization arising from nuclear deformation may enable the mechanical perception of the nucleus under restricted cell migration.

## 8. Discussion on Whether the Nucleus Functions as a Whole Mechanosensor That Controls Transcription and Alternative Splicing

### 8.1. Traditional View, the Entire Nucleus Acts as a Mechanosensor

**Figure 9:**
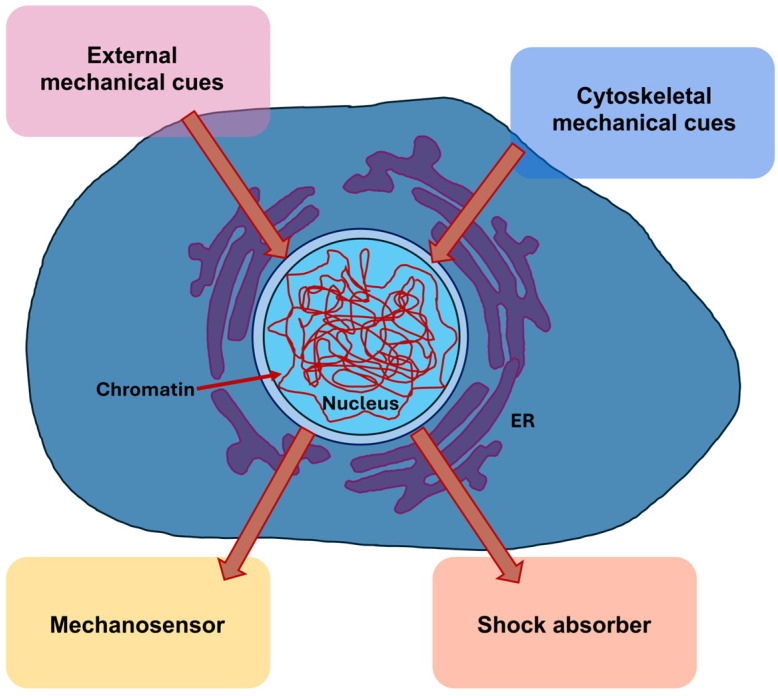
The cell nucleus functions as a mechanosensory and shock absorber. The cell nucleus is exposed to deformative forces (red arrows) when cells are compressed, stretched, or actively contracted due to external or internal forces. The cell nucleus responds to mechanical forces by perceiving them mechanically and then acting as a shock absorber so that the genetic material and associated components are not damaged.

In the following, the traditional view on the cell nucleus as a mechanosenor as a whole is discussed briefly. Mechanical signals for the cell nucleus include extrinsic forces (such as tissue stiffness and shear stress) and intrinsic forces (such as cell motility) that are conveyed to the cell nucleus through the cytoskeleton and NE, causing alterations in chromatin architecture, transcriptional activity, cell morphology, and even repair of DNA damage. This positions the cell nucleus as a powerful mechanosensor that converts physical cues into biochemical signals to direct the fate and functioning of the cell. The most important actors in this pathway are the LINC complex, the nuclear lamina, and actin filaments, which connect the external milieu to the genetic information contained within. How are mechanical cues transmitted the cell nucleus? They can be transmitted in a direct fashion. The forces from the ECM environment can propagate via the cytoskeleton, such as actin filaments and microtubules, to the NE and nuclear lamina. Thereby the LINC complex serves as a bridging element by physically coupling the interior of the nucleus with the cytoskeleton. Several processes known as mechanotransduction take place in the cell nucleus. First, there is the process of chromatin reorganization, in which forces stretch and remodel chromatin, changing its organization and thereby affecting gene accessibility. Second, there is the process of alterations in gene expression, in which chromatin modifications result in alterations in transcription, affecting cell fate, metabolic activity, and cellular differentiation. The third type involves processes that induce structural and shape alterations in the cell nucleus, enabling it to function as a shock absorber and alter nuclear shape (flattening, elongation) in order to adapt to the cell shape alterations. For example, actin dynamics play a critical role: Intranuclear actin polymerization is able to displace DNA damage repair regions, such as double-strand breaks, toward the nuclear periphery. The multicomponent architecture of the cell nucleus equips it to execute its various roles as a reaction vessel in the nanoscale level and as a mechanosensor and structural support in the microscale level (Figure 9). Moreover, due to its exceptional elastic characteristics and compressibility, the cell nucleus serves as a mechanosensor that captures dynamic variations in cell volume and functions as a mechanical shock absorber [111,140,194]. While the plasma membrane, cytoplasm, and related cytoskeleton are all extremely deformable, the cell nucleus is up to ten times stiffer than other parts of the cell, rendering its deformation a potentially rate-determining step and therefore a prime candidate for mechanosensing [219,222,378]. These mechanisms enable the cell nucleus to function both as a “shock absorber” and a “mechanosensory organelle,” which is important for keeping the cell working in reaction to physical changes in its microenvironment. Thus, an Elastic-Fluid Model for DNA damage and mutation has been proposed that is based on the nuclear fluid segregation during the migration of cells through a confinement [379]. The confinement during cell migration causes DNA damage and results in genomic variations. There is no evidence that these damages are caused by DNA breaks due to mechanical stress on the chromatin of the distorted cell nucleus.

This finding may be understood through the elastic-fluid model, according to which the cell nucleus can be viewed as an elastic-fluid system with an elastic element (chromatin) and a fluid element that can be pressed out when there is deformation of the cell nucleus. In addition, the elastic-fluidic model is linked to the kinetics of DNA strand breaks and reparations by postulating that the local volume fraction of the elastic element governs the amount of damage per unit volume arising from naturally encountered DNA strand breaks, while the volume fraction of the fluidic element governs the amount of DNA strand break repair per unit volume through repair enzymes that are dissolved in the surrounding liquid. Beyond that, the elimination of the fluid [380], and thus of the mobile DNA damage repair factors appears to be adequate to account for the amount of DNA injury and genomic variations noted in the experiments [107,379]. A similar elimination of transcription controllers or chromatin remodeling agents could impact on the activity of transcription.

**Figure 10:**
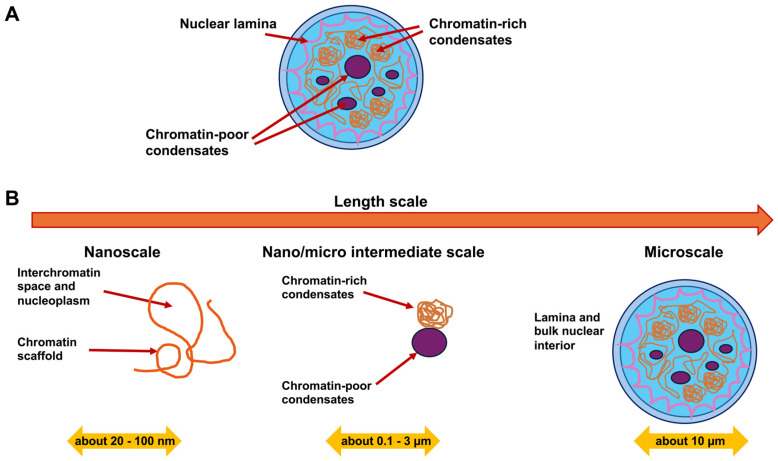
The cell nucleus is a multi-scale entity. (**A**) The cell nucleus consists of the nuclear lamina (magenta), which is located beneath the nuclear envelope (NE; pink). The interior of the cell nucleus comprises the chromatin network (orange) and biomolecular condensates, which are either chromatin-rich or chromatin-poor (dark orange). (**B**) The structure of the cell nucleus varies depending on the scale: at the nanoscale, the interior of the cell nucleus is divided into chromatin and the pore-filled interchromatin space. On the mesoscale, chromatin domains, biomolecular condensates, and nucleoli are discernible. On a few-micrometer scale, the cell nucleus can be viewed as a solitary organelle whose characteristics are determined by the nuclear lamina at its perimeter and all of its internal constituents.

Based on this organization, the structural, rheological, and mechanical characteristics of the nucleus vary according to the scale (Figure 10). Whereas small displacements or mechanical deformations in the nanometer range, equivalent to the pore size of the chromatin scaffold, are determined by the characteristics of the nucleoplasm fluid occupying the interchromatin voids, large displacements in the micrometer range or beyond, equivalent to the size of a nucleolus or chromosome territory, are influenced by the characteristics of these large nuclear constituents. Moreover, the size of the tracer particles employed to investigate the rheological or mechanical characteristics of the interior of the nucleus strongly influences the results. For instance, small tracers can be swapped between pores, while large tracers cannot and instead remain in a pore and track the movement of the chromatin scaffold [381]. As explained in the following, it is useful to evaluate the material characteristics of the cell nucleus and the connected biological processes in the setting of their corresponding temporal and spatial scales (Figure 10).

The cell nucleus can be characterized as a multi-scale heterogeneous viscoelastic system comprising three major elements with differing characteristics: nucleoplasmic fluid, chromatin scaffold, and nuclear lamina. All three elements have viscoelastic characteristics depending on the scale. The nucleoplasmic fluid can be regarded as largely fluid-like on either a small or large scale, whereas chromatin and lamina exhibit fluid-like and solid-like features. This behavior enables the cell nucleus to enable fast biochemical reactions on a nanoscale and to offer adjustable mechanical reinforcement for the cell on a microscale level. Nuclear proteins and RNAs not linked to chromatin are in the nucleoplasmic fluid that fills the inside of the porous chromatin framework, also known as the interchromatin space [157]. The interchromatin space actually takes up more space than the chromatin itself [161]. The fluid within it is heterogeneous, consisting of substructures like nucleoli and liquid-like condensates that are separate from the chromatin [382,383,384]. The observed pore size of the interchromatin space is approximately several tens of nanometers, as tracer particles exceeding this size exhibit a sharp decrease in their movement [385,386]. Finally, diffusion coefficients, viscosities, and stiffness coefficients measured on different length scales using varying experimental methods provide differing types of insights into the cell nucleus. This type of behavior is typical for heterogeneous multiscale viscoelastic matter. In conclusion, although there is substantial macromolecular crowding within the cell nucleus [387], the nucleoplasmic fluid occupying the interchromatin space can be viewed as a viscous fluid at the nanoscale, exhibiting a viscosity comparable to that of water. This enables biochemical reaction processes, like DNA-driven ones, to function efficiently, as their speed tends to slow down in a more viscous environment. In summary, it can be said that viewing the cell nucleus as a single mechanosensor appears too simplistic; therefore, a more detailed approach should be pursued that takes into account the various mechanotransduction mechanisms at different length scales. In addition to these largely direct effects of mechanotransduction, indirect aspects should also be discussed here, which are explained below.

### 8.2. Mechanotransduction-Induced Gene Expression and Alternative Splicing

The concept of the nucleus acting as a mechanosensory seems to be universal, as it has been reported for vastly different cell types. This causes extracellular and cytoplasmic forces to be transmitted via the NE to the interior of the cell nucleus, resulting in deformation of the chromatin and nuclear bodies [225,388]. Along with altering the positioning of genes or chromosomes, mechanical forces can directly change chromatin architecture and gene transcription. In vitro experiments demonstrate that a force of 5 pN is capable of decondensing individual chromatin fibers [389]. At low ionic strengths of 5 mM NaCl, three different force ranges can be identified in the elastic behavior of native chromatin fibers [389]. Between 0 and 7 pN, the strain-relaxation cycles produce reversible force–strain curves, which means that the curve recorded during relaxation matches the curve recorded during stretching, showing that the process is reversible and that the fiber is in equilibrium during the entire cycle. In this force range, the curves can also be repeated, i.e., the same fiber can be run through multiple times and will produce the same force–strain curves each time. More importantly, the force-stretch curves are monotonic and exhibit a gently positive curvature. In the medium force range (7–20 pN), the relaxation curve is no longer aligned with the strain curve (hysteresis) and only re-aligns with it below 2 pN. Nevertheless, the stretching and relaxation parts of the cycle remain consistently repeatable throughout consecutive cycles [389]. Another characteristic can be noticed between cycles that achieve varying maximum stretching forces. The elongation curves of a fiber stretched by different amounts in consecutive cycles are broadly similar in the common force regime, while the relaxation curves vary from cycle to cycle solely by a constant length factor. This suggests that fibers exposed to consecutive higher forces lengthen proportionally without altering their elastic characteristics. The hysteresis seen above 7 pN suggests that the speed at which the fiber is stretched or relaxed is faster than the speed at which the fiber stretches and contracts in equilibrium at this level of strain. Beyond 20 pN, the force–strain curves are no longer reversible or repeatable, which means that hysteresis occurs between each stretch and relaxation curve, and less force is required for each consecutive stretch to achieve an equivalent lengthening. When the fiber has been stretched beyond 20 pN, the consecutive stretch/relaxation curves of this fiber in the low force range no longer reflect the curves of fibers that have never been subjected to high forces. Between 20 and 35 pN, a transition occurs in the strain curve when the fiber is exposed to these forces for the first time. When stretching exceeds 20 pN, it is associated with irreversible fiber alteration, which may result in the loss of linker histones and histone octamers. When the tension approaches 65 pN, the transition characteristic of dsDNA upon overstretching can be seen [390].

The exertion of forces on the cell surface leads to immediate stretching of the chromatin in the cell nucleus, which is linked to a fast initiation of the transcription of a transgene present in this chromatin site [391]. It is noteworthy that the rate of transcription is strongly linked to the frequency and intensity of the forces exerted, and that interference with the LINC complex cancels out the force-driven transcription reaction [391]. The insight that force-triggered transcription proceeded at an exceptionally fast pace (less than 30 s) indicates that chromatin stretching is more likely to impact the transcriptional machinery’s accessibility to the gene or its transcriptional activity instead of the epigenetic condition of the gene locus. Although this is extremely fascinating, direct evidence of such modulation of gene expression for endogenous genes has yet to be confirmed. In addition, it will be interesting to find out if this gene transcription regulation mechanism just targets genes that are set up for transcription or if it can also turn on genes that are inactive, like those in heterochromatic locations. It is interesting to note that sustained force leads to an accumulation of heterochromatin and transcriptional silencing, which can be considered a mechanically adaptive response [181] and may function as a negative regulatory feedback loop. Finally, it is uncertain how force-triggered chromatin stretching could impart specific properties, as numerous genomic loci would likely be exposed to equivalent mechanical forces, and a direct connection between mechanoresponsive genes and LINC complex elements has not yet been identified. It seems plausible that transcription factors or other regulatory elements are influenced by the physical forces to which a cell and, subsequently, the cell nucleus are exposed. Moreover, it is assumed that post-transcriptional modification of genes, such as alternative splicing or 5′ capping of RNAs, play important roles in this process. In summary, it can be said that the application of force to the cell nucleus can cause chromatin stretching and promote the activation of a reporter transgene expression [391]. These results offer some of the most compelling evidence to support the idea that the cell nucleus is a mechanically responsive organelle.

Apart from the mechanotransduction-induced gene expression, the alternative splicing seems to be emerging fields in nuclear mechanotransduction [392]. Alternatively spliced molecules include ECM molecules like fibronectin, tenasin (TnC) and versican, the focal adhesion protein vinculin, Piezo1, Piezo2, and the potassium two-pore domain channel subfamily member K2 (synonymously referred to as Tandem of P domains in a Weakly Inward rectifying K^+^ channel (TWIK) or TREK-1) [392,393,394,395]. Moreover, it has been found that specifically, ECM proteins and focal adhesion proteins are alternatively spliced upon the exertion of forces toward the cell that all impact the adhesion and migration of cells. The question now arises as to how alternative splicing controls the functions of various molecules that trigger the mechanotransduction chain at the plasma membrane and the ECM. Mechanical cues can modify the activity, expression, or positioning of mechanosensitive splicing factors including RNA-binding fox-1 homologue 1 (RBFOX1), RBFOX2, SRSF1 and SRSF5, thereby conveying these mechanical variations [396]. For example, shear stress in endothelial cells controls the activities of specific RNA-binding proteins that govern the expression of splice variants [397]. RBFOX1 regulates the alternative splicing events of the pre-mRNAs of two key focal adhesion proteins, namely paxillin (*PXN*) and vinculin (*VCL*). In the absence of RBFOX1, *PXN* exon 6 and *VCL* exon 19 are spliced out, resulting in PXN and VCL proteins, while in the presence of RBFOX1, *PXN* exon 6 and *VCL* exon 19 are retained due to the binding of RBFOX1 to an (U)GGAUG motif located within the intron distal to these alternative exons. These alternative splicing variants lead to the generation of extended PXN (ePXN) and metavinculin (MVCL containing exon 19) proteins. Consequently, the alternative splicing of vinculin results in differences in actin filament binding between metavinculin and vinculin [398].

Recently, there has been a paradigm shift regarding the function of talin-1 (TLN1) in mechanosensation and mechanotransduction. TLN1 is primarily responsible for activating integrin receptors and conveying mechanical cues to the actin cytoskeleton through focal adhesions. Nevertheless, the distribution of TLN1 is not limited to focal adhesions. TLN1 resides in the cell nucleus in multiple human cell lines, wherein it is closely connected to chromatin [399]. It is crucial to note that the decrease in endogenous TLN1, which is caused by small interfering RNA (siRNA), results in broad alterations in the gene expression pattern of human breast epithelial cells. To assess the functional effect of nuclear TLN1, a TLN1 fusion protein encoding a nuclear localization signal is introduced. These results showed that the accumulation of nuclear TLN1 changes the expression of a subgroup of genes and affects the development of cell–cell clusters. This work offers an additional viewpoint on the canonical perspective of the subcellular localization and functionality of TLN1. Consequently, it can be concluded that the cells remodel both the environment and the molecules involved in mechanosensory and mechanotransduction, such as focal adhesion proteins, and even alter their localization. Another conclusion is that alternative splicing plays a crucial role in cancer invasion and metastasis, as has already been proven for osteosarcomas, Ewing sarcomas, chondrosarcomas, and various metastatic bone cancers, such as those originating in the prostate, bladder, and breast [400]. Alternative splicing not merely controls the proliferation, motility, and apoptosis of cancer cells, but also facilitates the transformation of both the tumor microenvironment and the bone metastatic niche [401]. Beyond alternative splicing, alternative polyadenylation takes place if there are multiple polyadenylation sites (PASs) present at the end of a transcript, which can result in 3′-untranslated regions (UTRs) of varying lengths. Alternative splicing and alternative polyadenylation substantially expand proteomic diversification and protein functionality within cells [402,403] and are critical in a multitude of pathophysiological conditions [403,404]. Alternative splicing and alternative polyadenylation are closely linked to each other and can be mutually dependent [405]. In mammalian cells, the last intron sequence at the 3′-end of an mRNA transcript (the terminal intron) is frequently eliminated following the formation of a polyadenylate (poly(A)) tail [405]. Recent research findings indicate that the splicing of the terminal intron could potentially be reliant on the length of the poly(A) tail [405].

## 9. Conclusions and Future Directions

Overall, characterization of the interface of the cell nucleus and cytoskeleton is still in its infancy, but there is promising potential for new molecular mechanisms in a number of critical processes that will further deepen our comprehension of the capabilities of this interface throughout the entire life cycle including diseases. It is assumed that alterations in the mechanosensory properties of the cell nucleus are likely to have a decisive role in the malignant transformation of cancer cells and the heterogeneity of cancers. Finally, the influence of the individual constituents of the cell nucleus on the mechanical modification of cell migration and invasion in cancer and its malignant progression has been discussed. It has been found that mechanical signals alter certain mechanosensory elements, including the entire cell nucleus, so that cells such as cancer cells can deform more easily and thus move more effectively through narrow passages. All mechanical alterations in the cell and cell nucleus must be captured so that the extent of the malignant progression of cancers can be analyzed and, in other cases, predicted when mechanomarkers are present. Although fundamental components such as nesprins and SUN proteins have been known for some time, recent studies indicate that the full intricacy of how physical forces control nuclear functionality and cell actions has only recently been explored. This interface serves as an important nodal point for the transmission of mechanical cues from the ECM scaffold to the genome and impacts various disease processes such as cancer. Gaining an appreciation of the dynamic control of LINC complex assembly, the function of actin polymerization inside the cell nucleus, and how these mechanisms fail in diseases like cancer holds exciting potential for new break throughs.

The various mechanosensory elements were evaluated by examining them on different length scales, for example, on nucleoli, nucleolar bodies, nuclear speckles or chromosomes, including nucleosomes. All known mechanobiological approaches are based on the hypothesis of a universal process of mechanosensation and mechanotransduction that is independent of cell types or tissues types. This has not yet been refuted and appears to be an excellent approach for managing the great diversity of processes. In particular, the transport of talin and integrins into the cell nucleus seems to be of great importance and has only recently been identified [406] or become more widely known. More comprehensive research is necessary at the functional level. A paradigm shift has taken place here: the view of the cell nucleus and its detailed structural elements depend on the length scale. All these mechanosensors must work together in a coordinated manner at the cellular level. They should work together hand in hand and rapidly transmit the mechanical signals to the cell nucleus, whereby the type of mechanical stimulus, its duration, and its repetition play a role.

Another major gap in this research area is the absence of efforts to link variations in mechanosensor activity to global alterations in transcription and RNA processing. Specifically, there are only a few models that capture mechanotransduction signaling routes from the moment a mechanical cue is picked up by a cell, through biochemical signaling, to the resulting alterations in gene expression that are controlled by RNA processing. Since mechanotransduction is involved in the homeostasis and function of numerous tissues and organ systems, future work will need to characterize the transcriptome and splicing profiles that respond to certain mechanical stimulations. In the future, biophysical investigations, cell biological analyses, and molecular biological analyses will be used together to represent the entire extent of changes in the cell nucleus and to show not only the direct changes but also the indirect ones.

## Figures and Tables

**Table 1:** Effect of small molecules and inhibitors on nuclear mechanical properties and mechanotransduction.

Compound/Inhibitor	Target	Effect on Cell Nucleus and Mechanotransduction
Actinomycin D	RNA Polymerase II	Represses nuclear blebbing
Betulinic acid	Lamin A/C	Reduces nuclear stiffness, supports motility
BIX01294	G9a	Decreases H3K9me2, elevates stiffness, decreases blebbing
Blebbistatin	Myosin-II	Decreases nuclear stress and deformation
Chaetocin	SUV39H1	Decreases H3K9me3, elevates nuclear blebbing, reduces stiffness
Leptomycin B	CRM (XPO1)	Impairs cargo transportation, alters signaling
Lonafarnib	Farnesyltransferase	Decreases nuclear stiffness in progeria
Metallo-proteinase (MMP) Inhibitors like Batimastat (BB-94), Marimastat (BB-2516), Prinomastat, and Ilomastat	Matrix-metalloproteinases	Impair ECM-facilitated signaling, decreased nuclear stiffness via indirectly impacting phosphorylation of lamin A/C
Nocodazole	Microtubules	Depolymerizes microtubules, impairs force transmission needed for nuclear positioning and shape preservation
Paclitaxel	Microtubules	Stabilizes microtubules, abnormal nuclear shape and micronucleation
Panobinostat (Pan-class I/II inhibitor)	HDAC	Increased nuclear irregularity, nuclear blebbing
Salermide (SIRT = class III inhibitor)	HDAC	Increased nuclear irregularity, nuclear blebbing
Selinexor (KPT-330)	CRM (XPO1)	Impairs cargo transportation, alters signaling
Y-27632	Rho-kinase (ROCK)	Impairs contractility, decreases nuclear stress and deformation

## Data Availability

No data was generated in this review article.

## Abbreviations

The following abbreviations are used in this manuscript:

5-LOX	5-lipoxygenase
AFM	Atomic force microscopy
AJs	Adherens junctions
ALT	Alternative lengthening of telomeres
AMPK	5′-AMP-activated protein kinase
APBB1IP	Amyloid beta precursor protein binding family B member interacting protein
APC	Adenomatous polyposis coli
AT1R	Angiotensin II type 1 receptor
ATP	Adenosine triphosphate
ATRX	α-thalassemia/intellectual disability syndrome X-linked
BAF	Barrier of autointegration factor
CaMKK2	Ca^2+^-calmodulin-dependent kinase kinase 2
CBs	Cajal bodies
cPLA2	Cytosolic phospholipase A2
CR	Cytoplasmic ring
CRP2	Cysteine and glycine-rich protein 2
DAXX	Death-domain associated protein
DJs	Digitation junctions
EAST	Expandable associated structure
ECM	Extracellular matrix
EDMD	Emery-Dreifuss muscular dystrophy
ENAH	Enabled homolog
EPCs	Epidermal stem/progenitor cells
ER	Endoplasmic reticulum
ESCRT	Endosomal sorting complexes required for transport
FAK	Focal adhesion kinase
FAs	Focal adhesions
FG	Phenylalanine-glycine
FHL2	Four-and-a-half LIM domains protein 2
FRET	Fluorescence resonance energy transfer
G4	G-quadruple
GEMs	Gemini of Cajal bodies
GPCRs	G protein-coupled receptor
H1R	Histamine H1 receptor
H3K27ac	Histone 3 lysine 27 acetylation
H3K9me2	Histone 3 lysine 9 dimethylation
H3K4me3	Histone 3 lysine 4 trimethylation
HDAC	Histone deacetylase
HIC-5	Hydrogen peroxide-inducible clone 5
HIRA	Histone cell cycle regulator
HMGB2	High mobility group B2
HMTs	Histone methyltransferases
INF2	Inverted formin-2
INM	Inner nuclear membrane
IR	Inner ring
Kuk	Kugelkern
LAP2	Lamina-associated polypeptide 2
LAPs	Lamin-associated proteins
LINC	Linker of nucleoskeleton and cytoskeleton
LLPs	Liquid–liquid phase separation
lnRNAs	Long non-coding RNAs
LPDs	Lipid packing defects
MAT	Mesenchymal-amoeboid transition
MeCP2	Methyl CpG binding protein 2
MIIA	Myosin-IIA
MIIB	Myosin-IIB
MRTF-A	Myocardin-related transcription factor-A
MSCs	Mesenchymal stem cells
NE	Nuclear envelope
NESs	Nuclear export signals
NLSs	Nuclear localization signals
NMMIIB	Non-muscle myosin IIB
NMMT	Nuclear membrane mechanotransduction
NPC	Nuclear pore complex
NR	Nuclear ring
NuMA	Nuclear mitotic apparatus
Nups	Nucleoporins
ONM	Outer nuclear membrane
PASs	Polyadenylation sites
PDE1	Phosphodiesterase 1
PDLIM7	PDZ and LIM domain 7
PDLSCs	Peridontal ligament stem cells
PGRL	Prostaglandin receptor-like
PML-NBs	Promyelocytic leukemia nuclear bodies
Poly(A)	Polyadenylate
PREP1	Pbx-regulating protein-1
RBDs	RNA-binding domains
RBFOX1	RNA-binding fox-1 homologue 1
RBPs	RNA-binding proteins
RIAM	Rap1 GTP-interacting adaptor molecule
RNPs	Ribonucleoprotein particles
ROCK	Rho-associated protein kinase
rRNA	Ribosomal RNA
SIMs	SUMO-interacting motifs
siRNA	Small interfering RNA
SMA	Spinal muscular atrophy
SMN	Survival motor neuron
snoRNPs	Small nucleolar RNPs
SUMO	Small ubiquitin-like regulator
TADs	Topologically associated domains
TAN	Transmembrane actin nuclear
TAZ	Transcriptional coactivator with PDZ-binding motif
TEMs	Tetraspanin-enriched microdomains
TGFb1I1	Transforming growth factor beta-1 induced transcript 1
TnC	Tenascin
TRP	Transient receptor potential
TRPV4	Transient receptor potential vanilloid 4
TWIK	Tandem of P domains in a weakly induced rectifying K^+^ channel
UTRs	3′-untranslated regions
VASP	Vasodilator-stimulated phosphoprotein
YAP	Yes-associated protein
